# Muscle atrophy‐related myotube‐derived exosomal microRNA in neuronal dysfunction: Targeting both coding and long noncoding RNAs

**DOI:** 10.1111/acel.13107

**Published:** 2020-03-31

**Authors:** Chia‐Pei Yang, Wan‐Shan Yang, Yu‐Hui Wong, Kai‐Hsuan Wang, Yuan‐Chi Teng, Ming‐Hsuan Chang, Ko‐Hsun Liao, Fang‐Shin Nian, Chuan‐Chuan Chao, Jin‐Wu Tsai, Wei‐Lun Hwang, Ming‐Wei Lin, Tsai‐Yu Tzeng, Pei‐Ning Wang, Mel Campbell, Liang‐Kung Chen, Ting‐Fen Tsai, Pei‐Ching Chang, Hsing‐Jien Kung

**Affiliations:** ^1^ Institute of Microbiology and Immunology National Yang‐Ming University Taipei Taiwan; ^2^ Brain Research Center National Yang‐Ming University Taipei Taiwan; ^3^ Institute of Molecular and Genomic Medicine National Health Research Institutes Zhunan Taiwan; ^4^ Program in Molecular Medicine School of Life Sciences National Yang‐Ming University and Academia Sinica Taipei Taiwan; ^5^ Department of Life Sciences Institute of Genome Sciences National Yang‐Ming University Taipei Taiwan; ^6^ Institute of Brain Science National Yang‐Ming University Taipei Taiwan; ^7^ Program in Molecular Medicine National Yang‐Ming University and Academia Sinica Taipei Taiwan; ^8^ The Ph.D. Program for Cancer Molecular Biology and Drug Discovery College of Medical Science and Technology Taipei Medical University Taipei Taiwan; ^9^ Department of Biotechnology and Laboratory Science in Medicine National Yang‐Ming University Taipei Taiwan; ^10^ Institute of Public Health National Yang‐Ming University Taipei Taiwan; ^11^ Cancer Progression Research Center National Yang‐Ming University Taipei Taiwan; ^12^ Department of Neurology Neurological Institute Taipei Veterans General Hospital Taipei Taiwan; ^13^ Aging and Health Research Center National Yang‐Ming University Taipei Taiwan; ^14^ UC Davis Comprehensive Cancer Center University of California Davis CA USA; ^15^ Department of Geriatric Medicine School of Medicine National Yang Ming University Taipei Taiwan; ^16^ Center for Geriatrics and Gerontology Taipei Veterans General Hospital Taipei Taiwan

**Keywords:** aging, HIF‐1α‐AS2, lncRNAs, miR‐29b‐3p, muscle atrophy

## Abstract

In mammals, microRNAs can be actively secreted from cells to blood. miR‐29b‐3p has been shown to play a pivotal role in muscle atrophy, but its role in intercellular communication is largely unknown. Here, we showed that miR‐29b‐3p was upregulated in normal and premature aging mouse muscle and plasma. miR‐29b‐3p was also upregulated in the blood of aging individuals, and circulating levels of miR‐29b‐3p were negatively correlated with relative appendicular skeletal muscle. Consistently, miR‐29b‐3p was observed in exosomes isolated from long‐term differentiated atrophic C2C12 cells. When C2C12‐derived miR‐29b‐3p‐containing exosomes were uptaken by neuronal SH‐SY5Y cells, increased miR‐29b‐3p levels in recipient cells were observed. Moreover, miR‐29b‐3p overexpression led to downregulation of neuronal‐related genes and inhibition of neuronal differentiation. Interestingly, we identified HIF1α‐AS2 as a novel c‐FOS targeting lncRNA that is induced by miR‐29b‐3p through down‐modulation of c‐FOS and is required for miR‐29b‐3p‐mediated neuronal differentiation inhibition. Our results suggest that atrophy‐associated circulating miR‐29b‐3p may mediate distal communication between muscle cells and neurons.

## Graphical abstract

miR‐29b‐3p‐containing exosomes released from atrophied muscle can be transported via the circulation and transferred to neuronal cells. Increased miR‐29b‐3p levels in neuronal cells may lead to inhibition of neuronal differentiation.

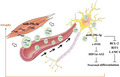

## 1 INTRODUCTION

According to the 2017 report from World Bank, a considerable portion of the worldwide population is of advanced age of 65 years and older (e.g., 27.0% in Japan, 13.9% in Korea, 21.5% in Germany, 18.5% in UK, and 15.4% in US populations). Aging is considered as one of the main risk factors for developing multiple afflictions, especially loss of muscle mass and neurodegeneration. Loss of muscle mass and strength referred to as sarcopenia is a leading cause of frailty that is highly related to a decline in the quality of life and an increase in medical care expenses (Doherty, 2003; Hedden & Gabrieli, 2004). Animal models that mimic physiological (Derave, Eijnde, Ramaekers, & Hespel, 2005; Kemp, Blazev, Stephenson, & Stephenson, 2009) or pathological (Holecek, 2012) muscle atrophy have been developed with the goal of identifying mechanisms associated with sarcopenia. For in vitro studies, the mouse C2C12 cell line developed by Yaffe and Saxel (1977) is one of the most well‐known systems used extensively in muscle atrophy research. It has been long known that catabolic factors such as glucocorticoid can cause muscle atrophy (Schakman, Gilson, & Thissen, 2008). Therefore, artificial glucocorticoid dexamethasone (Dex)‐treated cultured C2C12 myotubes have long served as an in vitro model system for muscle atrophy studies, a model more akin to the occurrence of sarcopenia. Emerging evidence points to the reciprocal communication between muscle and neuron, such that denervation results in muscle atrophy and muscle‐derived neurotrophic factors prevent motor neuron loss (Doherty, 2003). However, prior research has mainly focused on the direct effects on the neuromuscular junction. Little is known about muscle atrophy‐associated changes in modulating distal cell functions mediated via the circulation during the aging process.

MicroRNAs (miRNAs) with high expression in muscle have been identified by microarray and/or high‐throughput sequencing. Muscle‐specific miRNAs are involved in myogenesis (Horak, Novak, & Bienertova‐Vasku, 2016), and elevation of certain miRNAs in the circulation has been observed in muscle atrophy disorders (Coenen‐Stass, Wood, & Roberts, 2017). miRNAs are a family of small noncoding RNAs of 20‐22 nucleotides in length that directly target the 3'‐untranslated region (3'‐UTR) of mRNAs and repress gene translation (Wu & Belasco, 2008). Emerging evidence suggests that mature miRNAs can be actively secreted from histiocytes to blood. miRNAs circulate in the bloodstream as cargo of exosomes (Vickers, Palmisano, Shoucri, Shamburek, & Remaley, 2011) or complexed with protein factors (Arroyo et al., 2011). Exosomes are extracellular nanoparticles with a diameter of 40–150 nm (Koritzinsky, Street, Star, & Yuen, 2017) and important regulators of long‐range miRNA shuttling (Quattrocelli & Sampaolesi, 2015). Exosomes mediate cell‐to‐cell communication by transferring miRNAs to recipient cells and exert their inhibitory effects (Squadrito et al., 2014). Skeletal muscle is the largest tissue in the entire human body and comprises approximately 40% of body weight. Therefore, it is not surprising that sarcopenia‐associated upregulation of muscle miRNAs, which may be secreted to blood in large amounts, results in changes in miRNA profiles in plasma and/or serum during aging‐associated muscle atrophy. These miRNAs can be used as diagnostic biomarkers for aging‐associated muscle atrophy (Dhahbi, 2014). However, the role of circulating atrophy‐associated miRNAs in modulating neuronal cell functions has not been addressed.

We therefore hypothesize that miRNAs upregulated in atrophied muscle may be released and enter neuronal cells that consequently interfere with neuronal function. By using small RNA sequencing (smRNA‐seq) in combination with RT‐qPCR, we identify miR‐29b‐3p as a novel atrophy‐associated exosomal miRNA that is transferred to neuronal cells. The miR‐29 family (miR‐29a, miR‐29b, and miR‐29c) was initially identified as positive regulators of myogenesis and tumor suppressor in rhabdomyoblastoma (Wang et al., 2007). Many recent reports further showed that miR‐29b is increased in and contributes to multiple types of muscle atrophy. Here, we identified c‐FOS, BCL‐2, RIT1, and LAMC1 as neuronal differentiation‐related genes targeted by miR‐29b‐3p. miR‐29b‐3p expression is sufficient to inhibit neuronal cell differentiation. We also identify long noncoding RNA (lncRNA) HIF1α‐AS2 as a novel neuronal differentiation inhibitory RNA that is upregulated by miR‐29b‐3p through targeting c‐FOS. Finally, we demonstrate that HIF1α‐AS2 participates in miR‐29b‐3p‐mediated neuronal differentiation inhibition. These data suggest that miR‐29b‐3p may act as a communication mechanism from muscle to neuron which contributes to muscle atrophy‐induced neuronal dysfunction during aging.

## 2 RESULTS

### 2.1 miR‐29b‐3p, miR‐130b‐3p, and miR‐708‐3p are increased in plasma of aged mice and humans

**Figure 1:**
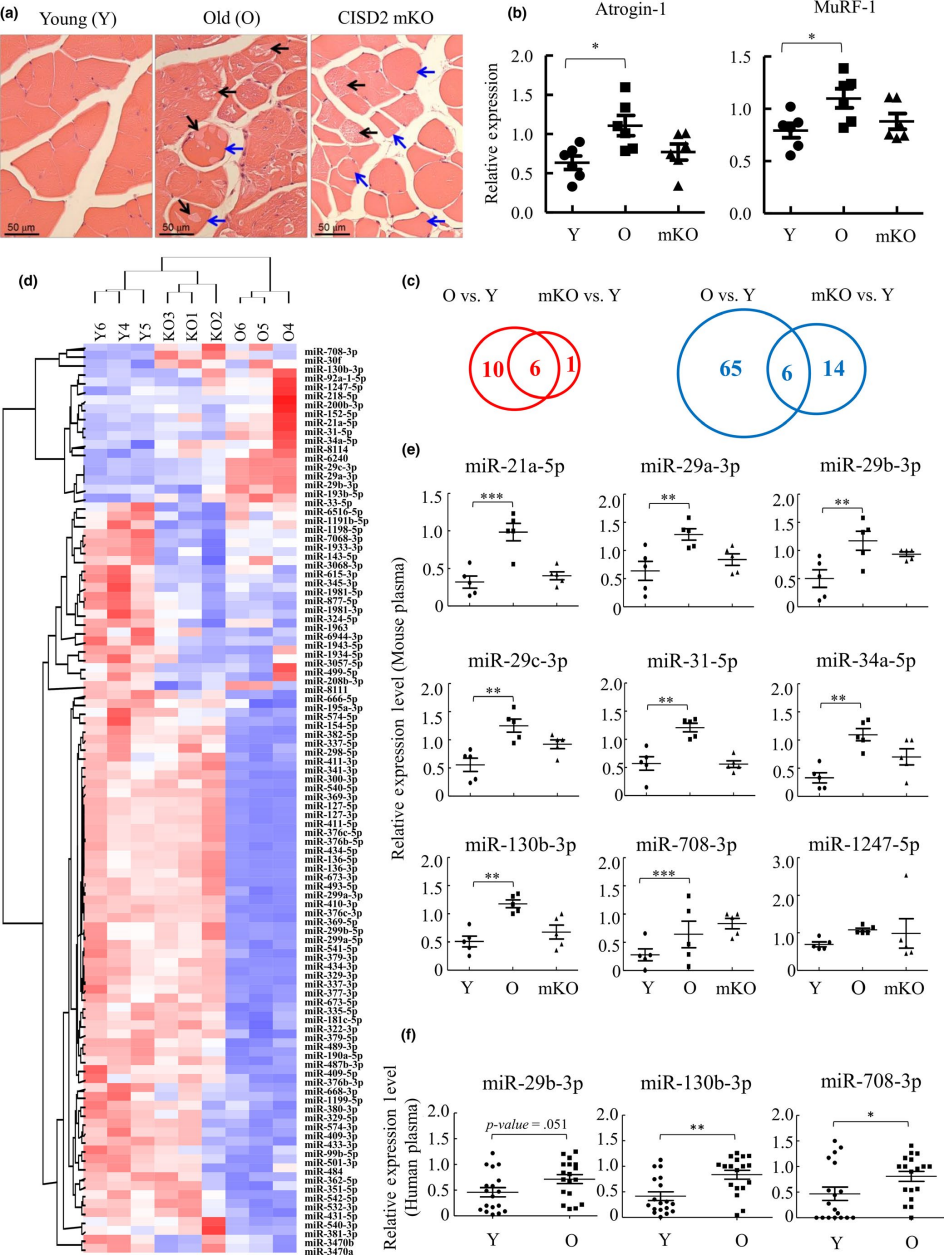
Identification of miRNAs that are increased in atrophied muscle and in plasma of aged mice and humans. (a) Hematoxylin–eosin (HE) staining of quadriceps femoris muscles from 3‐month (Young, Y)‐ and 26‐month (Old, O)‐old wild‐type mice and 3‐month‐old CISD2 muscle‐specific knockout (mKO) mice revealed degeneration (black arrows) and cellular shrinkage (blue arrows) in the skeletal muscles of naturally aged and CISD2 mKO mice. (b) RT‐qPCR analysis of Atrogin‐1 and MuRF‐1 mRNA using femoris muscle RNA isolated from mice described in (a) (*n* = 6). (c) Venn diagram showing the number of the miRNAs up (left panel, red)‐ and down (right panel, blue)regulated in aged and CISD2 mKO muscle (RPKM > 1, fold change > 1.5x) (*n* = 3). (d) Hierarchical clustering of the 102 miRNAs shown in (c). Each column represents the miRNA expression in Y, O, and CISD2 mKO muscle. (e) Plasma RNA isolated from Y, O, and CISD2 mKO mice (*n* = 5) was subjected to RT‐qPCR. 9 miRNAs were detected in mouse plasma. (f) Plasma RNA isolated from young (21–30 years old) and old (71–80 years old) human subjects (*n* = 18) was subjected to RT‐qPCR. miR‐708‐3p and miR‐130b‐3p were significantly increased in plasma of elderly, and miRNA‐29b‐3p was increased with borderline statistical significance (*p* = .051). *p < .05, **p < .01, ***p < .001 by one‐way ANOVA

To identify miRNAs upregulated during aging‐associated muscle atrophy, both normal and premature aging mouse models were used. For natural aging, 26‐month‐old wild‐type mice with 10%–20% gray hair indicating depigmentation of the cloth were used. For premature aging, we used 3‐month‐old CDGSH iron–sulfur domain‐containing protein 2 (CISD2) muscle‐specific knockout (mKO) mice with early depigmentation and gray hair (Huang et al., 2018). CISD2 is a mitochondrial outer membrane protein (MOMP) that participates in maintaining mitochondrial integrity, and its deficiency drives premature aging (Chen, Kao, Chen, et al., 2009; Chen, Kao, Kirby, & Tsai, 2009). Conversely, persistent expression of CISD2 extends life span and delays aging in the mouse model (Wu et al., 2012). Moreover, our recent report showed that CISD2 mKO mice shared similar proteomic profiling as naturally aged mice (Huang et al., 2018). These data together make CISD2 mKO mice an ideal premature aging model. Following hematoxylin–eosin (HE) staining that confirmed the degeneration (Figure 1a, black arrows) and cellular shrinkage (Figure 1a, blue arrows) of femoris muscles and elevation of muscle atrogenes Atrogin‐1 and MuRF‐1 (Figure 1b), the hallmarks of muscle atrophy, in naturally aged and CISD2 mKO mice, small RNA sequencing (smRNA‐seq) was performed on the femoris muscle from three young (3 months, Y), old (26 months, O), and muscle‐specific CISD2 KO mice (3 months, mKO). Expression levels of 102 miRNAs were found to display a fold change greater than 1.5 with statistical significance (*p* < .05) in old and CISD2 mKO mice as compared to young mice (Figure 1c–d).

We then asked whether the increased expression of femoris miRNAs is reflected by their contents in plasma as potential biomarkers. Among the 17 miRNAs that were upregulated in femoris of either aged or CISD2 mKO mice, 9 were detectable in mouse plasma by RT‐qPCR and 8 were upregulated in old mice when compared with young mice (Figure 1e). In CISD2 mKO mice, 4 miRNAs (miR‐29b‐3p, miR‐29c‐3p, miR‐34a‐5p, and miR‐708‐3p) were increased with borderline statistical significance (*p* = .076, *p* = .057, *p* = .075, and *p* = .075, respectively) (Figure 1e). Among these 9 miRNAs, miR‐708‐3p and miR‐130b‐3p were also significantly upregulated in old human subjects when compared with young (21–30 years old) (Figure 1f). Moreover, miRNA‐29b‐3p was increased with borderline statistical significance (*p* = .051). Interestingly, Spearman correlation showed that miR‐29b‐3p and miR‐130b‐3p are significantly correlated with certain sarcopenic factor(s) (Table 1). This result indicates that a small subset of aging‐associated miRNAs are secreted from muscle tissue and protected in the circulation, presumably by their interaction with proteins or embedded in microvesicles, such as exosomes.

**Table 1:** Spearman correlation coefficients (*r*) and *p*‐value (*p*) between the levels of miR‐29b‐3p, miR‐708‐3p, and miR‐130b‐3p and various sarcopenic factors

	miR−29b−3p		miR−708−3p		miR−130b−3p	
	*r*	*p*	*r*	*p*	*r*	*p*
Education	.209	.376	−.068	.790	.297	.231
Grip strength	.005	.984	−.279	.262	.186	.460
Walking speed	−.172	.469	−.005	.984	−.276	.268
LBM	−.369	.110	.035	.889	**−.603**	**.008**
ASM	−.416	.068	.023	.928	**−.611**	**.007**
RASM	**−.499**	**.025**	−.082	.746	**−.730**	**.001**
MMSE	−.048	.840	.217	.387	.161	.523

Abbreviations: LBM, lean body mass; ASM, appendicular skeletal muscle; RASM, relative ASM; MMSE, mini‐mental state examination.

Bold indicating the correlation between the sacropenic factors and microRNA expression is medium to high (r > 0.3 or r < ‐0.3) and the p‐value is statistically significant (p < 0.05).

### 2.2 Prolonged horse serum treatment induces C2C12 myotube atrophy and upregulation of miR‐29b‐3p

The mouse C2C12 cell line developed by Yaffe and Saxel (1977) differentiates into myotube when shifted to serum‐free medium containing horse serum (Bains, Ponte, Blau, & Kedes, 1984; Sultan, Henkel, Terlou, & Haagsman, 2006) and has been used extensively in muscle‐related research. Dexamethasone (Dex)‐induced C2C12 myotube atrophy is one of the well‐known in vitro model systems for muscle atrophy that is akin to sarcopenia (Bodine et al., 2001; Gomes, Lecker, Jagoe, Navon, & Goldberg, 2001). One recent report performed detailed dose‐ and time‐dependent studies and showed that 10 μM of Dex treatment for 24 hr induced significant atrophy in C2C12 myotubes, as evidenced by a decrease in myotube diameter and myosin heavy chain (MyHC) content. Moreover, the expression of muscle atrogenes Atrogin‐1 and MuRF‐1 was elevated at 3 hr after Dex treatment.

To evaluate whether prolonged horse serum treatment of C2C12 represents another suitable in vitro myotube atrophy model of age‐associated sarcopenia, we compared the C2C12 myotube diameter on days 6, 8, and 12 after 5% exosome‐free horse serum treatment. Our data showed that C2C12 cells displayed approximately 90% myotube phenotype on day 6 and exhibited a significant reduction in myotube diameter on days 8 and 12 (Figure S1a‐b). Similar to aged mouse muscle in vivo (Figure 1b), the expression of muscle atrogenes Atrogin‐1 and MuRF‐1 was continuously increased, but to a less extent than Dex treatment (Figure S1c). However, MyHC content did not show a significant change during this experimental period (Figure S1d). These data suggest the prolonged horse serum treatment leads to a gradual induction of muscle atrophy when compared to Dex treatment. Since age‐associated muscle atrophy is a slow‐progressing process compared to cachexia, we therefore used prolonged horse serum treatment of C2C12 myotubes as an in vitro model to study the role of identified miRNAs in muscle atrophy. Following treatment of C2C12 myoblasts with 5% horse serum for 6 and 8 days, the expression of miR‐29b‐3p, miR‐708‐3p, and miR‐130b‐3p was analyzed. miR‐29b‐3p was the only miRNA whose level was increased in C2C12 cells upon prolonged horse serum treatment‐induced myotube atrophy (Figure S1e). Its level was also increased after Dex treatment for 6 days. Therefore, miR‐29b‐3p was chosen for further studies.

### 2.3 miR‐29b‐3p is not a downstream target of CISD2

Given the role of CISD2 (Huang et al., 2018) and miR‐29b‐3p (Li, Chan, et al., 2017) in muscle atrophy and upregulation of plasma miR‐29b‐3p in CISD2 mKO mice (Figure 1e), we hypothesize that CISD2 may directly or indirectly upregulate miR‐29b‐3p in atrophied C2C12 myotubes. To study this, we first generated a CISD2 KO C2C12 cell line using CRISPR/Cas9n system (Figure S2a‐c). Given the role of CISD2 in mitochondrial functions, we first measured mitochondrial respiration in control and CISD2 KO C2C12 cells using a Seahorse bioanalyzer. We showed that CISD2 depletion did not influence the mitochondrial content (Figure S2d), basal respiration, or ATP production, but only reduced the spare respiration capacity (Figure S2e). However, knockout CISD2 significantly inhibited the differentiation of C2C12 myoblasts (Figure S2f). Since CISD2 KO C2C12 cells can no longer differentiate, we used shRNA to knockdown CISD2 in C2C12 myotubes following 5% horse serum treatment for 6 days. We found that the level of miR‐29b‐3p was not upregulated in partial CISD2 knockdown C2C12 myotubes at day 8 of differentiation (Figure S3a). We further studied exosomal miR‐29b‐3p levels in CISD2 knockdown C2C12 myotubes. Exosomal miR‐29b‐3p was also not altered by CISD2 knockdown (Figure S3b). These data suggest that the expression of miR‐29b‐3p may not be regulated by CISD2. Unknown mechanisms requiring factors in the context of the whole‐animal environment may be needed for miR‐29b‐3p upregulation upon aging.

### 2.4 Transfer of myotube secreted exosomal miR‐29b‐3p to neuronal cells

**Figure 2:**
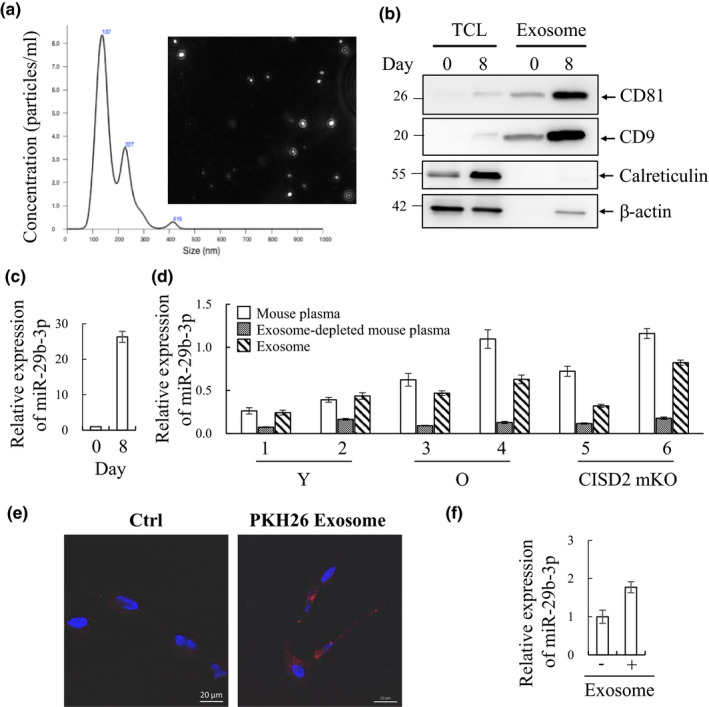
C2C12 myotube‐derived exosomes are taken up by RA‐differentiated SH‐SY5Y cells and results in increased level of miR‐29b‐3p. (a) Exosomes isolated by differential ultracentrifugation were analyzed using nanoparticle tracking analysis (NTA) and represented as size versus. concentration. (b) Immunoblotting assessing the exosome markers, CD81 and CD9, in C2C12 myotube‐derived exosomes enriched from undifferentiated (day 0) or 8 days differentiated cells. The exosome fraction is absent of endoplasmic reticulum (ER) marker calreticulin. β‐Actin was used as loading control. TCL, total cell lysate. (c) Exosomal RNA was purified by TRIzol, and miR‐29b‐3p quantity was determined by RT‐qPCR. (d) RNA from mouse plasma, exosome, and exosome‐depleted plasma was purified by TRIzol, and miR‐29b‐3p quantity was determined by RT‐qPCR. (e) SH‐SY5Y cells were induced for differentiation with 10 μM retinoic acid (RA) for 72 hr. RA‐differentiated SH‐SY5Y cells were maintained in the absence (left) or presence (right) of PKH26‐labeled exosomes (red). 24 hr after co‐incubation, cells were fixed, stained, and visualized by confocal (63x) microscope, demonstrating the presence of exosomes within cells. (f) RNA from RA‐differentiated SH‐SY5Y cells co‐cultured with or without long‐term differentiated C2C12 myotube‐derived exosomes was purified by TRIzol, and miR‐29b‐3p quantity was determined by RT‐qPCR. Error bars show mean ± *SD* (*n* = 3)

Since it has been shown that miRNAs are secreted from muscle cells via exosomes (De Gasperi et al., 2017), we first isolated exosomes from large volumes of C2C12 supernatants with and without horse serum‐induced differentiation for 8 days using differential ultracentrifugation. Nanoparticle tracking analysis (NTA) showing the modal size of the vesicles purified from supernatants was 137 nm, which were within the typical size of exosomes (<150 nm) (Figure 2a) (Tkach & Thery, 2016). Furthermore, immunoblotting confirmed the vesicles expressed conventional exosome markers, CD81 and CD9, but lacked the endoplasmic reticulum (ER) marker calreticulin that should not be present in exosomes (Figure 2b). Exosomal RNAs were purified, and RT‐qPCR analysis showed the level of exosomal miR‐29b‐3p was significantly higher in prolonged horse serum‐induced C2C12 myotubes (Figure 2c). To confirm the miR‐29b‐3p existed in exosomes of mouse plasma, we first purified miR‐29b‐3p from plasma, exosomes purified from plasma, and exosome‐depleted plasma from young, old, and CISD2 mKO mice using a commercial exosome isolation kit. RT‐qPCR analysis of miR‐29b‐3p showed it was reduced in exosome‐depleted plasma when compared with its plasma and exosomal levels (Figure 2d). Consistent with our hypothesis, these data demonstrate miR‐29b‐3p as exosomal cargo.

It is well recognized that exosomes are secreted from various types of cells, circulate in body fluids, and alter the function of the recipient cells through delivery of exosomal cargo including miRNAs (Zhang et al., 2015). To investigate whether the miR‐29b‐3p‐carrying exosomes secreted from muscle cells can be uptaken by neuronal cells, the receipt of C2C12 myotube‐derived exosomes by a human neuroblastoma cell line SH‐SY5Y cells that are often used as in vitro model of neuronal function and differentiation was examined. To study this, 20 µg/ml of PKH26‐labeled myotube exosomes (Red; TRITC) was incubated with SH‐SY5Y cells, after 10 µM retinoic acid (RA)‐induced differentiation for 3 days. 24 hr after incubation, cells were fixed with 4% paraformaldehyde and stained with DAPI. Confocal fluorescence microscopy showed intracellular co‐localization of PKH26‐positive exosomes within cells (Figure 2e). Subsequently, we investigated whether myotube exosomes altered the miR‐29b‐3p levels in RA‐treated SH‐SY5Y cells. Consistent with our hypothesis, 48 hr after co‐culture of myotube exosomes with RA‐treated SH‐SY5Y cells, a significant increase in miR‐29b‐3p within SH‐SY5Y cells was identified (Figure 2f). These findings suggest that miR‐29b‐3p upregulated in atrophied muscle cells may be released and subsequently uptaken by neuronal cells possibly resulting in interference with the neuronal function.

### 2.5 c‐FOS, BCL‐2, RIT1, and LAMC1 are target genes of miR‐29b‐3p

It has been long proposed that skeletal muscle‐derived neurotrophic factors may be important for the survival of motor neurons. We therefore used three bioinformatics tools to identify whether 3′‐UTRs of neurotrophic factors contain potential miR‐29b‐3p target sites. Based on at least two prediction software packages, we were able to identify the miR‐29b‐3p target sites in 3′‐UTRs of 5 neurotrophic factors (Table S3, Bold). The RT‐qPCR analysis showed that the expression of 4 of these factors was detectable in C2C12 cells. However, their expression levels were not significantly changed at days 6 and 8 of horse serum‐induced C2C12 myotubes (Figure S4a) and upon miR‐29b‐3p overexpression (Figure S4b). This result indicates that neurotrophic factors, tested thus far, are not affected by miR‐29b‐3p and suggests that potential neuronal function of miR‐29b‐3p is probably not mediated through modulating the expression of neurotrophic factors.

**Figure 3:**
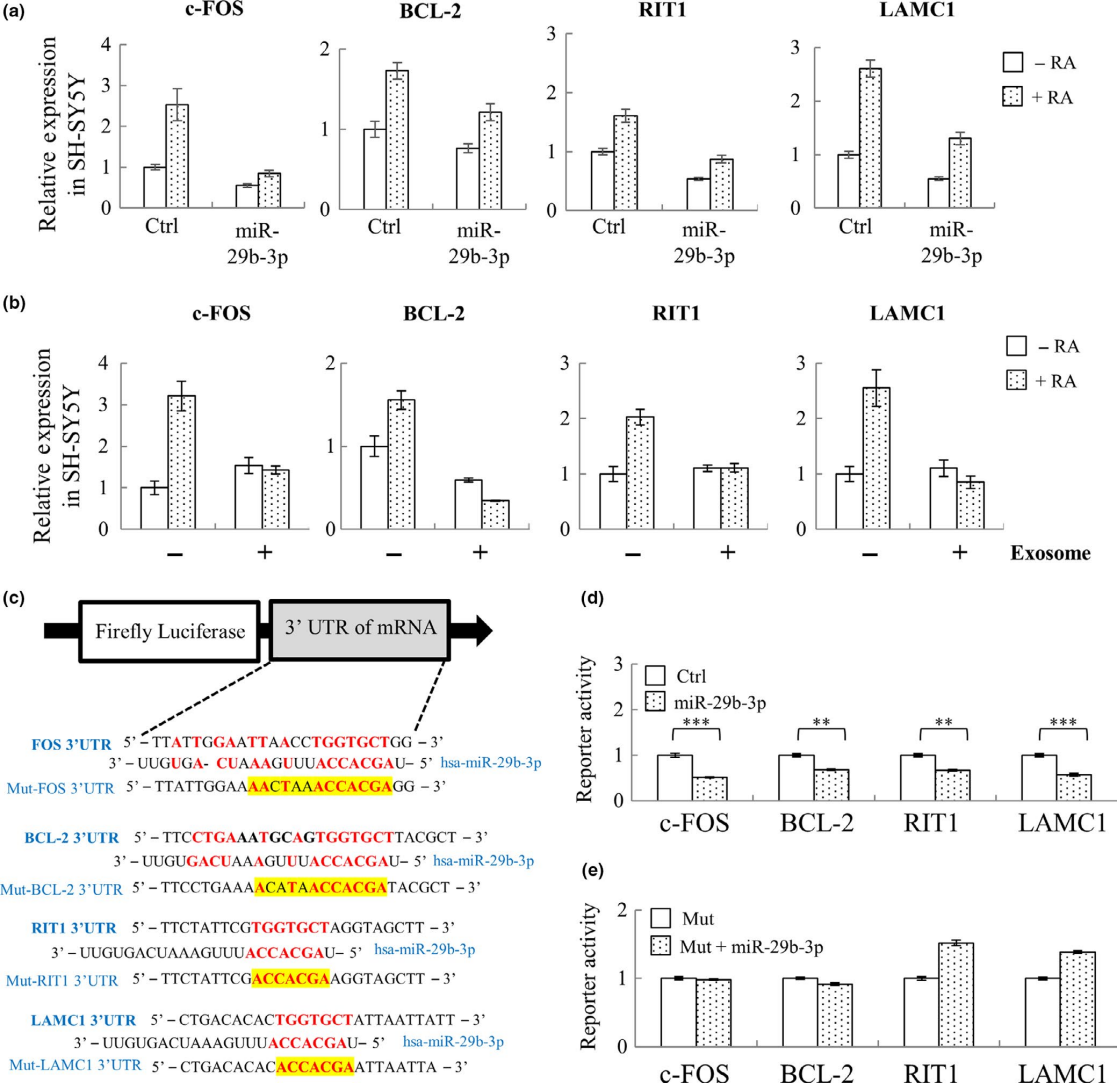
miR‐29b‐3p directly targets neuronal‐related genes c‐FOS, BCL‐2, RIT1, and LAMC1. (a) SH‐SY5Y cells were transiently transduced with lentivirus carrying control or pLenti4‐CMV/TO‐miR‐29b‐3p vector. 48 hr after transduction, SH‐SY5Y cells were treated with 10 μM RA for another 72 hr, followed by total RNA isolation and RT‐qPCR quantification of c‐FOS, BCL‐2, RIT1, and LAMC1. (b) SH‐SY5Y cells were treated with exosome for 24 hr and then induced for differentiation with 10 μM RA for 72 another hours. RNA from RA‐differentiated SH‐SY5Y cells co‐cultured with or without long‐term differentiated C2C12 myotube‐derived exosomes was purified, followed by quantification of c‐FOS, BCL‐2, RIT1, and LAMC1 using RT‐qPCR. (c) Structure of the luciferase reporter construct and the predicted miR‐29b‐3p binding site on the 3’UTR of c‐FOS, BCL‐2, RIT1, and LAMC1. (d and e) The luciferase reporter plasmids containing either miR‐29b‐3p binding site (d) or miR‐29b‐3p binding‐deficient mutant (Mut) (e) were co‐transfected with miR‐29b‐3p expression construct into 293T cells. Luciferase reporter assay results showing that c‐FOS, BCL‐2, RIT1, and LAMC1 were direct targets of miR‐29b‐3p. Error bars show mean ± *SD* (*n* = 3). **p < .01, ***p < .001 by *Student's t* test

Having established that miR‐29b‐3p does not affect the expression of a select number of neurotrophic factors present in C2C12 cells, we proceeded to investigate alternative mechanisms by which miR‐29b‐3p may modulate neuronal function. We downloaded 1732 neuronal‐related genes from Ingenuity Pathway Analysis (IPA) software and used the three bioinformatics tools to identify whether their 3′‐UTR contains potential miR‐29b‐3p target sites. Among them, we were able to identify miR‐29b‐3p target sites in the 3′‐UTR of 19 genes by at least two prediction software packages (Table S4). The expression level of these 19 candidates was screened in SH‐SY5Y cells. The expression of DNMT3B, GSK3B, IFNG, and MYCN was undetectable in the cells. Of the remaining 15 predicted targets, successful repression of c‐FOS, BCL‐2, RIT1, and LAMC1 by miR‐29b‐3p was observed in RA‐treated SH‐SY5Y cells (Figure 3a). Consistently, co‐culture of C2C12 myotube exosomes with RA‐treated SH‐SY5Y cells significantly repressed the levels of c‐FOS, BCL‐2, RIT1, and LAMC1 (Figure 3b). The direct repression of c‐FOS, BCL‐2, RIT1, and LAMC1 by miR‐29b‐3p was explored using a luciferase reporter assay. The 3'‐UTR of c‐FOS, BCL‐2, RIT1, and LAMC1 containing the seed sequences of miR‐29b‐3p was inserted downstream of luciferase cDNA (Figure 3c). Luciferase assays showed that exogenous overexpression of miR‐29b‐3p reduced the luciferase activity of each construct (Figure 3d). This repression of luciferase activity was reversed when the binding site for miR‐29b‐3p in 3'‐UTR of target genes was mutated (Figure 3e). These results suggest that c‐FOS, BCL‐2, RIT1, and LAMC1 are direct targets of miR‐29b‐3p.

### 2.6 miR‐29b‐3p inhibits differentiation of neuronal cells

**Figure 4:**
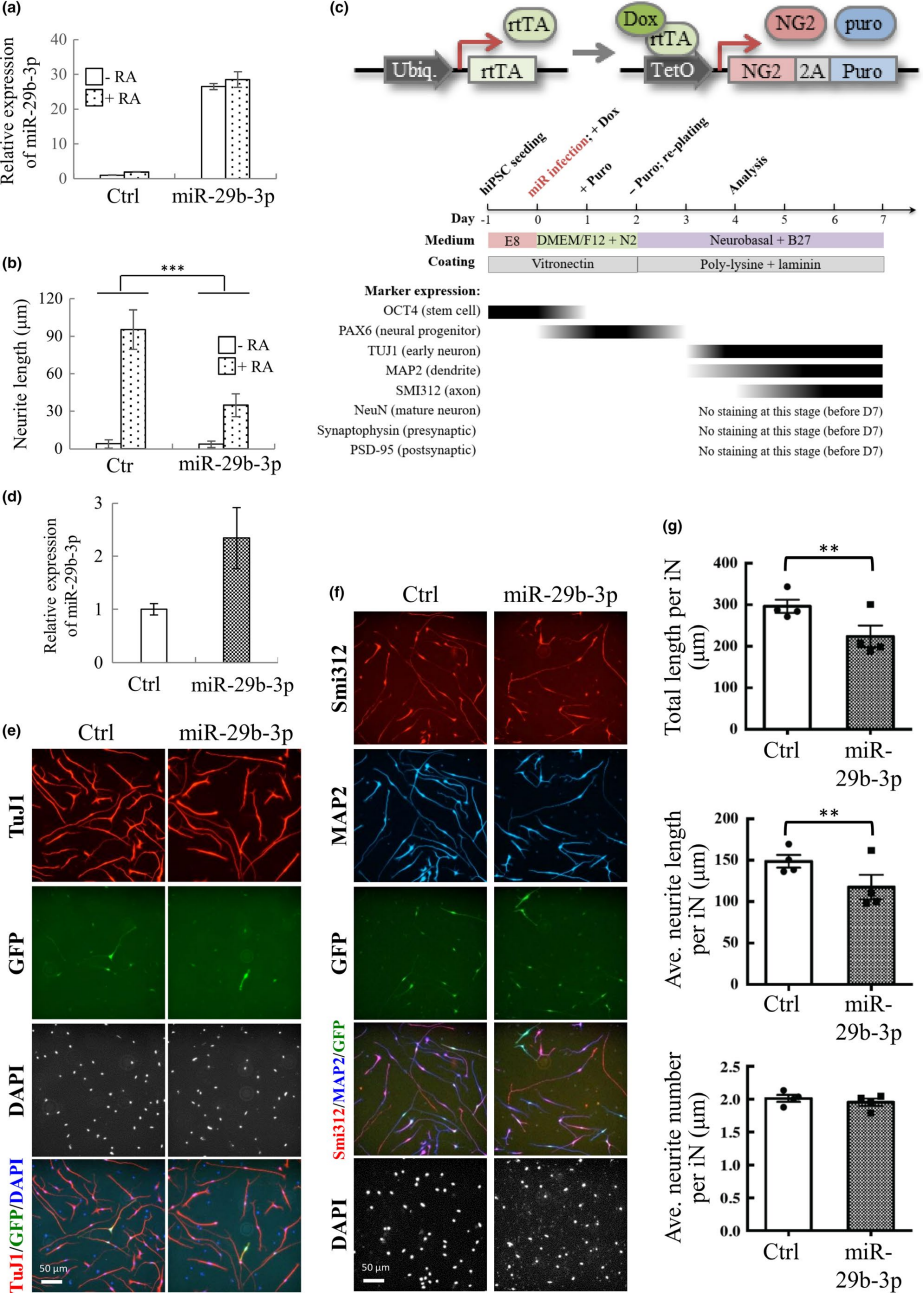
miR‐29b‐3p inhibits SH‐SY5Y and iNs’ cell differentiation. (a) SH‐SY5Y cells were transiently transduced with lentivirus carrying control or pLenti4‐CMV/TO‐miR‐29b‐3p vector. 48 hr after transduction, SH‐SY5Y cells were treated with 10 μM RA for another 72 hr, followed by total RNA isolation and RT‐qPCR quantification of miR‐29b‐3p. (b) SH‐SY5Y cells treated as described in (a) were stained with cell membrane and nucleus and used for quantification of neurite length. (c) Vector design for NG2‐mediated conversion of hiPSCs into glutamatergic neurons (upper panel). Flow diagram depicting the workflow involved in the generation of glutamatergic neurons (middle panel). hiPSCs were sequentially infected with lentivirus‐expressing rtTA and NG2‐puromycin resistance fusion protein linked by T2A sequence. Following lentivirus transduction and Dox treatment, hiPSCs were selected by puromycin for 24 hr and then re‐seeded for analysis. Time course of biomarkers expression following iNs’ induction is shown (lower panel). (d) Total RNA isolation and RT‐qPCR quantification of miR‐29b‐3p on day 3. (e and f) Representative images of iNs on day 4. The iN cells infected with control and miR‐29b‐3p construct were fixed and immunostained with anti‐TuJ1 (e, red), anti‐MAP2 (f, blue), and anti‐Smi312 (f, red) antibodies. (g) Quantification of the total length (upper panel), average neurite length (middle panel), and average neurite number (lower panel) of TuJ1‐positive neurites in GFP‐positive cells. Data are presented as mean ± *SEM* (*N* = 4). ***p < .001 by two‐way ANOVA (b) and *p < .05, **p < .01 by *Student's t* test of (g)

BCL‐2 (Akhtar, Ness, & Roth, 2004), RIT1 (Shi, Cai, & Andres, 2013), and LAMC1 (Cao, Pfaff, & Gage, 2007) are well‐known neuronal differentiation regulators. Therefore, we first probed the role of miR‐29b‐3p in neuronal differentiation. To this end, SH‐SY5Y cells transduced with control or lentivirus‐expressing miR‐29b‐3p were subjected to differentiation by treatment with RA. Successful overexpression of miR‐29b‐3p in undifferentiated and RA‐treated SH‐SY5Y cells was first confirmed by RT‐qPCR (Figure 4a). The degree of neuronal differentiation was visualized by staining SH‐SY5Y cells with cell membrane stain (neurite outgrowth kit, Thermo), photographed, and quantified based on the average neurite length using MetaMorph (Molecular Devices), which automatically defined the cell bodies and neurite extensions. The cellular differentiation of SH‐SY5Y was successfully induced by RA in control cells based on the increase in average neurite length (Shipley, Mangold, & Szpara, 2016) and the neuronal differentiation efficiency by RA appeared to be significantly suppressed by miR‐29b‐3p overexpression when compared with control (Figure 4b).

To further validate the inhibitory effect on neuronal differentiation by miR‐29b‐3p in a nonmalignant setting, human induced pluripotent stem cells (hiPSCs) were generated and differentiated into neurons (iNs) using a neurogenin‐2 (NG2) induction method (Zhang et al., 2013). Briefly, hiPSC/NG2 was first generated by transduction of hiPSCs with lentiviruses that constitutively express rtTA and tetracycline‐inducible (tetO promoter) expression of NG2 (Figure 4c, upper panel). The expression of neuronal markers was first examined, and the results are summarized in the lower panel of Figure 4c. To study the function of miR‐29b‐3p, hiPSCs/NG2 was further transduced with lentivirus‐expressing control or miR‐29b‐3p together with lentivirus‐expressing GFP, and then subjected to differentiation by treating with doxycycline (Dox). Successful overexpression of miR‐29b‐3p in hiPSCs/NG2 was confirmed by RT‐qPCR (Figure 4d). To access the neuronal differentiation, we stained iNs with neuronal markers at 4 days after induction. The iNs displayed the pan‐neuronal marker (TuJ1) (Figure 4e), a somatodendritic marker (MAP2), and an axonal marker (Smi312) (Figure 4f). Consistent with our results in SH‐SY5Y cells, overexpression of miR‐29b‐3p decreased not only the total length of TuJ1^+^ neurites in iNs (296.51 ± 15.97 μm for the mock control, vs. 223.96 ± 25.95 μm for miR‐29b‐3p‐infected iNs) (Figure 4g, upper panel), but also the average length of TuJ1^+^ neurites in iNs (148.78 ± 7.62 μm for the mock control, vs. 117.58 ± 15 μm for miR‐29b‐3p‐infected iNs) (Figure 4g, middle panel), whereas the average number of neurites per iN was not different between two groups (Figure 4g, lower panel). Consistently, co‐culture of iNs with 20% human plasma from elderly subjects with sarcopenia resulted in a decreased average length of neurites in iNs as well as the number of cells with neurite outgrowth, when compared to cells cultured with healthy control serum (Figure S5). These results suggest that miR‐29b‐3p upregulated during aging and can affect neuronal differentiation. To provide in vivo evidence, we further stained brain section of young (3 months) and old (25 months) mice with neuronal nuclei marker NeuN, somatodendritic marker MAP2, and DAPI. Consistently, the staining results showed that the length of the apical dendrites was significantly reduced in the cerebral cortex of old mice when compared to young mice (with relatively low plasma miR‐29b‐3p level when compared with old mice) (Figure S6).

### 2.7 HIF1α‐AS2 is a novel neuronal differentiation‐related long noncoding RNA (lncRNA) co‐upregulated by miR‐29b‐3p overexpression

**Figure 5:**
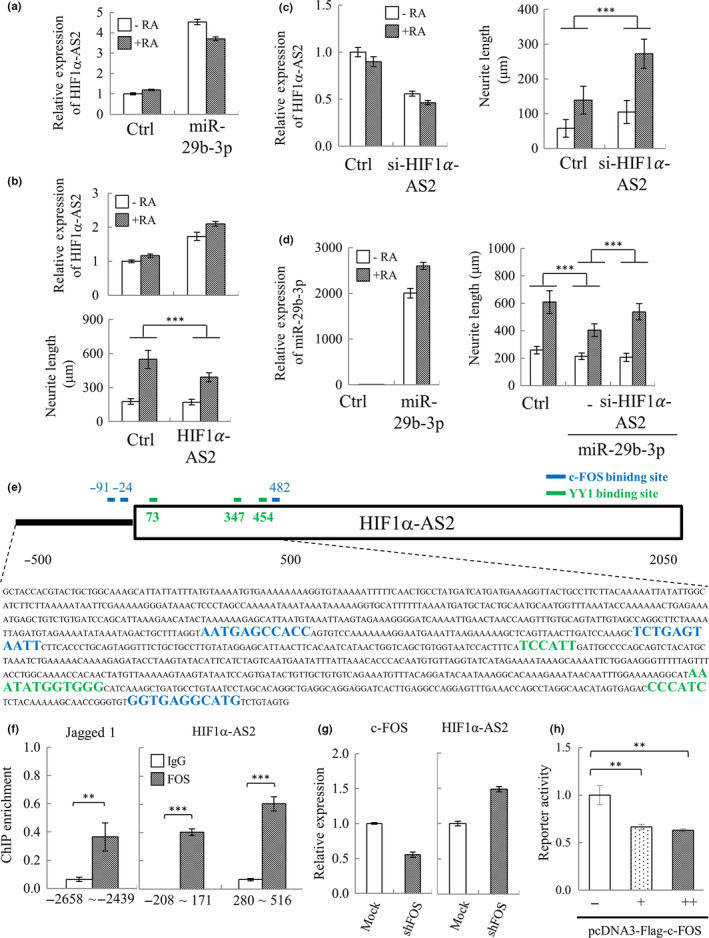
Identification of HIF1**α**‐AS2 as a novel miR‐29b‐3p co‐upregulated lncRNA that negatively modulates RA‐induced SH‐SY5Y differentiation. (a) SH‐SY5Y cells were transfected with pre‐miR‐29b‐3p. 24 hr after transfection, cells were treated with 10 μM RA for another 72 hr. Total RNA purified from cells was subjected to lncRNA qPCR array analysis. Array result for HIF1α‐AS2 is shown. (b) SH‐SY5Y‐HIF1α‐AS2 and control cells were treated with 10 μM RA for 72 hr. The levels of HIF1α‐AS2 were measured in both RA‐treated and RA‐untreated cells by RT‐qPCR (upper panel). SH‐SY5Y cells were stained as described in Figure 4b, and neurite length was quantified (lower panel). (c) SH‐SY5Y cells were transiently transfected with siRNA specific for HIF1α‐AS2 (si‐HIF1α‐AS2). 24 hr after transfection, the cells were treated with 10 μM RA for another 72 hr. HIF1α‐AS2 levels (left panel) and neurite length (right panel) were quantified as described in (b). (d) SH‐SY5Y cells were co‐transfected with pre‐miR‐29b‐3p and si‐HIF1α‐AS2. 24 hr after transfection, cells were treated with 10 μM RA for 72 hr. The expression level of miR‐29b‐3p (left panel) and neurite length (right panel) was quantified as described in (b). (e) Schematic representation of the putative c‐FOS binding sites in HIF1α‐AS2 promoter (TSS ± 500 bp) predicted by JASPAR database. (f) ChIP‐qPCR analysis using anti‐c‐FOS‐specific antibody revealed direct binding of c‐FOS to the promoter region of HIF1α‐AS2 (right panel). Jagged 1 promoter is the positive control of ChIP (left panel). (g) SH‐SY5Y cells were transiently transduced with lentivirus carrying control or pLKO.1‐shFOS (TRCN0000016004) vector. 72 hr after transduction, knockdown of c‐FOS (left panel) and expression of HIF1α‐AS2 (right panel) were detected by RT‐qPCR. (h) The luciferase reporter plasmid containing HIF1α‐AS2 promoter was co‐transfected with pcDNA3‐Flag‐c‐FOS into 293T cells. Luciferase reporter assay results showing that c‐FOS directly targets the HIF1α‐AS2 promoter. Error bars show mean ± *SD* (*n* = 3). **p < .01, ***p < .001 by *Student's t* test (f), one‐way ANOVA (h), and two‐way ANOVA (b, c and d)

In addition to protein coding RNAs, a wide variety of lncRNAs have recently been discovered. To identify the miR‐29b‐3p‐targeted lncRNAs that are involved in neuronal differentiation, we first used our lncRNA qPCR array (Shih et al., 2017) to screen the potential miR‐29b‐3p targets in SH‐SY5Y cells. We identified 8 lncRNAs whose levels are altered following miR‐29b‐3p overexpression. Among them, CDKN2B‐AS1, KRASP1, and NOS2P2 were downregulated by miR‐29b‐3p overexpression in control but not RA‐treated SH‐SY5Y cells. For the other 4 lncRNAs, MEG3 was upregulated in control and MALAT1, MIR31HG, and PCGEM1 were upregulated in RA‐treated SH‐SY5Y cells after miR‐29b‐3p overexpression (data not shown). Interestingly, HIF1α‐AS2 was the only lncRNA screened that is significantly upregulated in both control and RA‐treated SH‐SY5Y cells upon miR‐29b‐3p overexpression (Figure 5a). Therefore, following the lncRNA qPCR array screening, HIF1α‐AS2 was chosen for further experiments.

To elucidate the functional role of HIF1α‐AS2 in neuronal differentiation, we assayed the effect of HIF1α‐AS2 gain and loss of function on neurite outgrowth using control and RA‐treated SH‐SY5Y cells. For the ectopic expression experiment, we first generated a constitutive HIF1α‐AS2 overexpressing cell line by transient transfection of an expression plasmid for HIF1α‐AS2 into SH‐SY5Y followed by antibiotic selection for 2 weeks. Overexpression of HIF1α‐AS2 was identified in both control and RA‐treated SH‐SY5Y cells (Figure 5b, upper panel). Overexpression of HIF1α‐AS2 significantly inhibited the neurite outgrowth of RA‐treated SH‐SY5Y cells (Figure 5b, lower panel). Conversely, for the loss‐of‐function study, synthetic siRNA specific for HIF1α‐AS2 was transiently transfected into control and RA‐treated SH‐SY5Y cells. Significant knockdown of HIF1α‐AS2 was first confirmed in SH‐SY5Y cells (Figure 5c, left panel). Knockdown of HIF1α‐AS2 significantly increased the neurite outgrowth in RA‐treated SH‐SY5Y cells (Figure 5c, right panel). The potential of HIF1α‐AS2 in mediating miR‐29b‐3p‐induced inhibition of SH‐SY5Y differentiation was demonstrated by knockdown of HIF1α‐AS2 partially rescuing miR‐29b‐3p overexpression (Figure 5d, left panel) inhibited RA‐induced neurite outgrowth (Figure 5d, right panel). Together, these results indicate that HIF1α‐AS2 is a critical effector of miR‐29b‐3p and that the miR‐29b‐3p‐HIF1α‐AS2 axis is important for inhibition of neuronal differentiation.

### 2.8 c‐FOS mediates the upregulation of HIF1α‐AS2 upon miR‐29b‐3p overexpression

To explore what triggers the miR‐29b‐3p‐mediated upregulation of HIF1α‐AS2, we first analyzed the promoter region (transcription start site, TSS ± 500bp) of HIF1α‐AS2 by JASPAR (http://jaspar.genereg.net/), an open‐access database of nonredundant transcription factor (TF) binding profiles (Khan et al., 2018), and identified 3 potential c‐FOS binding sites (Figure 5e). To analyze the binding of c‐FOS on HIF1α‐AS2 promoter, we performed a chromatin immunoprecipitation (ChIP) assay of c‐FOS using chromatin prepared from SH‐SY5Y cells. TSS −208 ~ 171 containing 2 potential c‐FOS binding sites and TSS 280 ~ 516 containing the third c‐FOS binding site were chosen for quantitative PCR (qPCR) analysis. Jagged 1 promoter region containing the AP‐1 site, a well‐known target of c‐FOS, was used for positive control. ChIP‐qPCR results showed that c‐FOS significantly binds on the promoter regions of HIF1α‐AS2 (Figure 5f). c‐FOS is known to cause gene activation as well as repression (Zhang, Chan, Sanchez‐Guerrero, & Khachigian, 2012). It has been shown that in complex with YY1, c‐FOS is able to recruit HDAC and repress gene expression (Zhang et al., 2012). Interestingly, we also identified 3 potential YY1 binding sites adjacent to the c‐FOS binding sites on HIF1α‐AS2 promoter (Figure 5e). To elucidate the role of c‐Fos in transcriptional regulation of HIF1α‐AS2, we analyzed the expression levels of HIF1α‐AS2 in SH‐SY5Y cells after knockdown of c‐FOS. RT‐qPCR analysis showed that HIF1α‐AS2 was increased upon knockdown of c‐Fos (Figure 5g). Moreover, the direct repression of HIF1α‐AS2 by c‐FOS was further explored using a luciferase reporter assay. To this end, the promoter region of HIF1α‐AS2 was cloned upstream of the luciferase gene. Luciferase assays showed that exogenous overexpression of c‐FOS reduced the promoter activity of HIF1α‐AS2 (Figure 5h). Together, our data showed that direct targeting c‐FOS by miR‐29b‐3p may be a potential mechanism that leads to the upregulation of HIF1α‐AS2 upon ectopic expression of miR‐29b‐3p.

## 3 DISCUSSION

Sarcopenia is characterized by the age‐related loss of muscle mass (atrophy) and strength (frailty) in humans (Cruz‐Jentoft AJ et al., 2010). Aging‐related sarcopenia that leads to progressive disability is a major healthcare issue worldwide. Emerging evidence shows that the levels of circulating small noncoding RNAs, especially miRNAs, are significantly changed during aging and muscle atrophy, and hold high potential to serve as biomarkers (Coenen‐Stass et al., 2017; Dhahbi, 2014). Since muscle is the largest tissue of human body, we hypothesized that miRNAs upregulated in atrophied muscle may be released from muscle cells to body fluids that consequently result in increased levels of atrophy‐associated miRNAs in plasma. These miRNAs are potential biomarkers for the detection of aging‐related sarcopenia. Following this hypothesis, we examined the miRNA profiles in muscle tissue of young (3 months), old (26 months), and CISD2 mKO mice, a premature aging mouse model. Following smRNA‐seq, we identified 17 miRNAs that were upregulated in aged muscle (Figure 1c). Among these, 3 were significantly higher in plasma of elderly human subjects (Figure 1f), suggesting their being aging‐related muscle atrophy‐associated miRNAs that are secreted from muscle cells to the bloodstream.

Here, we observed an interesting phenomenon in which the prolonged treatment of C2C12 myotubes with 5% horse serum induces a decrease in myotube diameter, an indicator of atrophy. Atrophying muscles show increased protein degradation via activating the ubiquitin (Ub) proteasome pathway. To further evaluate whether the prolonged horse serum treatment induces C2C12 myotube atrophy, we analyzed the expression of Atrogin‐1 and MuRF‐1, two well‐known muscle‐specific Ub‐ligases that are increased in a variety of muscle atrophy conditions (Bodine et al., 2001; Gomes et al., 2001). Our data showed that prolonged horse serum treatment induced a lower extent of upregulation of Atrogin‐1 and MuRF‐1 when compared with Dex treatment (Figure S1c). Since age‐associated muscle atrophy is a slow‐progressing process compared to cachexia, we therefore suggest that prolonged horse serum treatment of C2C12 myotubes may become a suitable in vitro model to study age‐associated muscle atrophy. By using this in vitro model system, we narrowed down the muscle atrophy‐associated miRNA candidates to miR‐29b‐3p (Figure S1e). Together with our finding that miR‐29b‐3p was increased in both aged mouse muscle tissue and plasma of aged mice and humans (Figure 1e‐f), suggestive of miR‐29b‐3p being secreted from age‐atrophied muscle into the circulation. However, the pathophysiologic role of these atrophy‐associated circulating miRNAs in aging remains unclear. Circulating miRNAs is either complexed with protein factors or enclosed in exosomes (Schwarzenbach, Nishida, Calin, & Pantel, 2014). To analyze whether atrophied myotube‐secreted exosomes contain miR‐29b‐3p, we purified exosomes from the supernatants of C2C12 myoblasts and long‐term differentiation‐induced atrophied C2C12 myotubes. Consistent with our hypothesis, the level of miR‐29b‐3p was significantly higher in exosomes purified from C2C12 myotubes after 8 days of differentiation when compared to control (Figure 2c). Moreover, a significantly higher quantity of plasma exosomal miR‐29b‐3p was observed when compared with miR‐29b‐3p levels in exosome‐depleted plasma (Figure 2d). These data indicate that miR‐29b‐3p is in exosomes and may therefore be available for uptake by other tissues, such as neurons, or act on muscle tissue in an autocrine manner.

**Figure 6:**
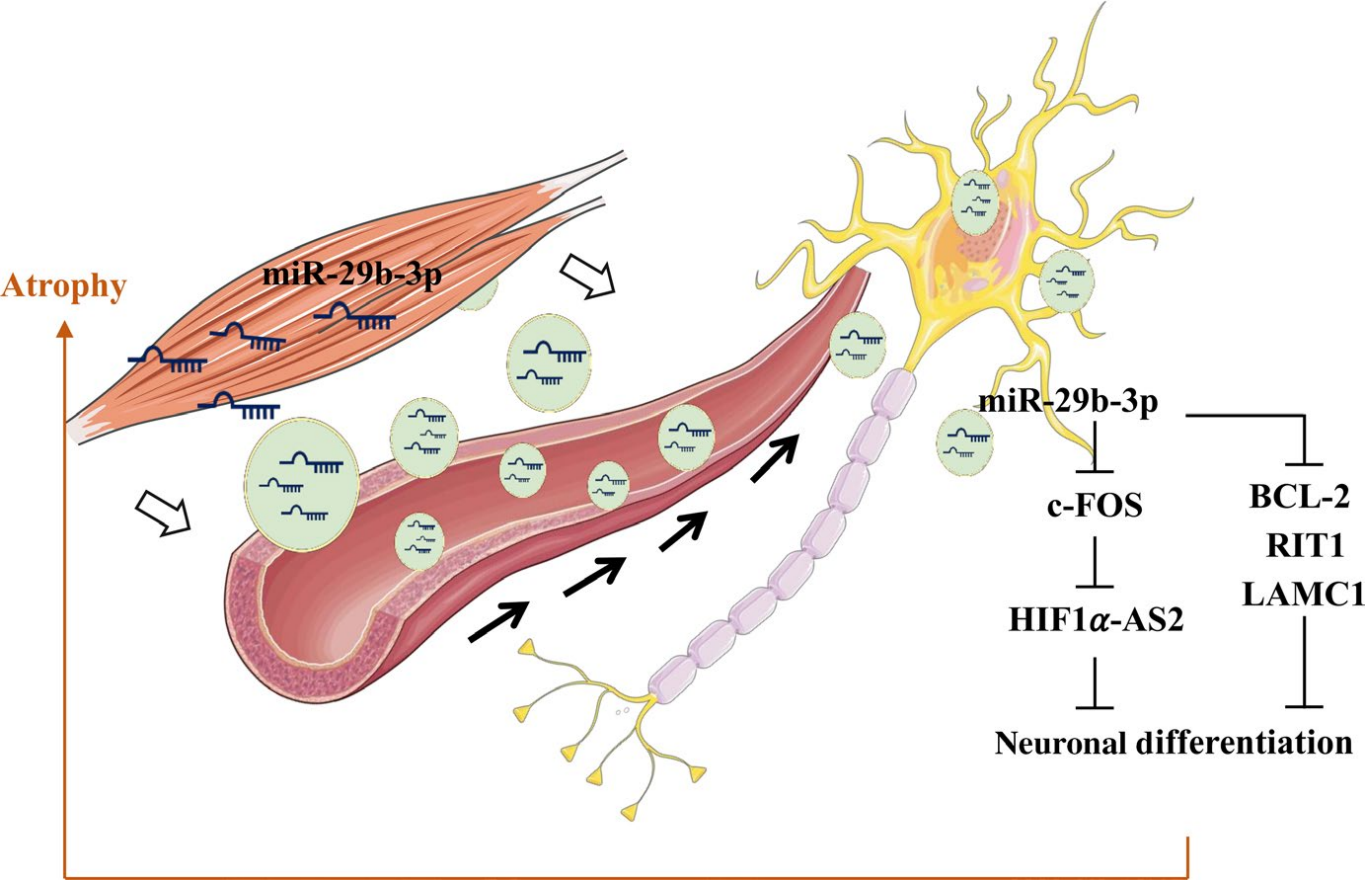
A schematic model of myotube‐derived exosomal miR‐29b‐3p in modulating neuronal cell function. miR‐29b‐3p‐containing exosomes released from atrophied muscle can be transported via the circulation and transferred to neuronal cells. Increased miR‐29b‐3p levels in recipient cells lead to (i) downregulation of neuronal differentiation‐related genes BCL‐2, RIT1, and LAMC1, and (ii) downregulation of c‐FOS, de‐repression of HIF1α‐AS2, and consequently inhibition of neuronal differentiation. The model depicts how atrophy‐associated exosomal miR‐29b‐3p may mediate distal communication between muscle and neuronal cells

It has been long known that denervation will cause muscle atrophy. Reciprocal regulation between muscle and neuron may exist during the aging process. However, it is largely unknown. Here, we showed the potential for uptake of myotube exosomes by neuronal SH‐SY5Y cells (Figure 2e) and a consequent increase in miR‐29b‐3p in these cells (Figure 2f). Interestingly, in addition to regulating myocyte function through targeting IGF‐1 and PI3K (p85α) (Li, Chan, et al., 2017), miR‐29b was also found to function in neuronal cells, though opposite responses were observed. During neuronal maturation, miR‐29b contributes to the inhibition of apoptosis through targeting proapoptotic BH3‐only genes Bim, Bmf, Hrk, Puma, and N‐Bak (Kole, Swahari, Hammond, & Deshmukh, 2011). In contrast, in mature neuronal cells, miR‐29b promotes neuronal cell death by modulating the expression of Bcl2L2 (Shi et al., 2012). Having shown that C2C12 myotube‐derived exosomes can transfer miR‐29b‐3p to SH‐SY5Y cells, we subsequently investigated the role of miR‐29b‐3p in modulating neuronal function. Targets we identified were c‐FOS, a neuronal activity marker (Chandra & Lobo, 2017), BCL‐2, a well‐known anti‐apoptotic protein that participates in neuronal differentiation (Akhtar et al., 2004), RIT1, a well‐known regulator in neuronal differentiation (Shi et al., 2013), and LAMC1, a neuronal differentiation regulator (Cao et al., 2007) (Figure 3). These results imply miR‐29b‐3p may inhibit neuronal differentiation through targeting these genes. We therefore overexpressed miR‐29b‐3p in SH‐SY5Y cells (Figure 4a) and found that miR‐29b‐3p overexpression significantly inhibited RA‐induced SH‐SY5Y differentiation (Figure 4b). Employing hiPSC technology, we generated an alternative in vitro model system to confirm our results in immortalized SH‐SY5Y cells. In this system, hiPSC‐derived neurons (iNs) were generated via NG2 induction. Importantly, overexpression of miR‐29b‐3p decreased the average length of neurites in iNs (Figure 4c‐g). Moreover, co‐culture of iNs with sarcopenic human plasma slightly decreased neurite outgrowth of iNs (Figure S5). These results together suggest that atrophied muscle may impair the function of neurons through exosomal miRNAs (Figure 6). Moreover, derangements in innervation may consequently affect the integrity of neuromuscular junctions, thus promoting the occurrence of muscle atrophy (Figure 6). These are interesting topics worth further exploration in the future.

Interestingly, other than mRNAs, we identified HIF1α‐AS2 as a novel neuronal differentiation‐related lncRNA (Figure 5b‐c) that is induced by miR‐29b‐3p (Figure 5a). Recent studies showed that HIF1α‐AS2 is dysregulated in various types of cancer (Chen et al., 2016; Jiang et al., 2016) and it may function as a competing endogenous RNA (ceRNA) to regulate the target mRNA of miRNA (Li, Wang, et al., 2017). Virtually nothing is known about the functions of HIF1α‐AS2 in neuronal differentiation. Here, we showed that overexpression of miR‐29b‐3p increased the level of HIF1α‐AS2 in both control and RA‐treated SH‐SY5Y cells (Figure 5a). The knockdown of HIF1α‐AS2 partially reversed the miR‐29b‐3p‐mediated blockade of neuronal differentiation (Figure 5d), indicating it is an effector of miR‐29b‐3p. However, other factors, such as the miR‐29b‐3p targeting neuronal differentiation regulators c‐FOS, BCL‐2, RIT1, and LAMC1 that we identified in this study (Figure 3), could also be involved. RIT1, a Ras subfamily GTPase, is known to regulate neuronal differentiation via ERK and p38 pathways (Shi et al., 2013) and neurogenesis via Akt/Sox2 pathway (Mir, Cai, & Andres, 2017). LAMC1 (laminin C) is an extracellular matrix protein contributing to the adhesion and survival of neural cells undergoing differentiation (Sun et al., 2008). Most importantly, we found that c‐FOS binds to HIF1α‐AS2 promoter and represses its expression (Figure 5e‐h). Direct targeting c‐FOS by miR‐29b‐3p may be one potential mechanism of how miR‐29b‐3p upregulates HIF1α‐AS2. Our discovery here warrants further investigation.

In summary, we identified miR‐29b‐3p as a muscle atrophy‐associated exosomal miRNA with potential to be uptake by neuronal cells which consequently inhibits neuronal cell differentiation via targeting the mRNAs of c‐FOS, BCL‐2, RIT1, LAMC1, and upregulation of lncRNA HIF1α‐AS2. These data suggest that miR‐29b‐3p might serve as a potential biomarker for muscle atrophy and targeting miR‐29b‐3p could represent a potential therapeutic approach for muscle atrophy‐induced neuronal dysfunction.

## 4 EXPERIMENTAL PROCEDURES

### 4.1 Cell culture

The C2C12 and NTUH‐iPSC‐02‐02 were purchased from Bioresource Collection and Research Center of Food Industry Research and Development Institute, Taiwan. SH‐SY5Y cell line was kindly provided by Dr. L.S. Kao (National Yang‐Ming University, Taiwan). Cell culture conditions and generation of cell lines are described in Supporting Methods.

### 4.2 Human specimens

The study of human specimens at old age (71–80 year) has been approved by the Institutional Review Board (IRB) of Taipei Veterans General Hospital (2011‐04‐011 and 2015‐10‐001A). The study of human specimens at young age (21–30 year) has been approved by the IRB of National Yang‐Ming University (YM102021). All subjects analyzed were male.

### 4.3 Mice

CISD2 muscle‐specific KO (mKO) mice were generated as previously described (Huang et al., 2018). The mice were bred in a specific pathogen‐free facility. The animal protocols were approved by the Institutional Animal Care and Use Committee of National Yang‐Ming University. All mice analyzed were male with a C57BL/6 background.

### 4.4 Transfection of small‐interfering RNA (siRNA)

SH‐SY5Y cells were seeded at 2 ~ 5 × 10^4^ cells/well of 6‐well plates. The following day, the cells were transfected with 10 μmol scramble siRNA (scRNA) or siRNA specific for HIF1α‐AS2 (Ambion) using Lipofectamine^®^ RNAiMAX Reagent (Invitrogen) according to the manufacturer's instructions.

### 4.5 RNA extraction and reverse transcription and quantitative polymerase chain reaction (RT‐qPCR)

Detailed methods are provided in Appendix S1.

### 4.6 Small RNA sequencing (smRNA‐seq) and data analysis

Detailed methods are provided in Supporting Methods. The sequencing results were analyzed by using an in‐house bioinformatics pipeline (Cheng et al., 2013).

### 4.7 Isolation of exosomes

For miRNA detection, exosomes were isolated using the Total Exosome Isolation Reagent (Invitrogen). To prepare a large quantity of exosomes, a differential ultracentrifugation method was carried out as described previously (Bhome et al., 2017).

### 4.8 Nanoparticle tracking analysis (NTA)

The size distribution of exosomes was assessed by NTA (NS300; NanoSight, Amesbury, UK) that was equipped with a cCMOS camera and a 488‐nm blue laser. The instrument software (NTA 3.2.16) was used to perform the analysis.

### 4.9 Fluorescent labeling and transfer of exosomes

Exosomes were labeled with lipophilic cell tracking dye PKH26 (Sigma). Detailed methods are provided in Supporting Methods.

### 4.10 Luciferase reporter assays

Detailed methods are provided in Appendix S1.

### 4.11 Mitochondrial staining and oxygen consumption assay

Detailed methods are provided in Appendix S1.

### 4.12 Immunofluorescence (IF) staining

Detailed methods are provided in Appendix S1.

### 4.13 Immunoblotting assay

Detailed methods are provided in Appendix S1.

### 4.14 Chromatin Immunoprecipitation (ChIP) and real‐time quantitative PCR (qPCR)

ChIP was performed according to the protocol from Dr. Farnham's laboratory (http://genomics.ucdavis.edu/farnham). ChIP DNA was quantified by real‐time qPCR. Sequence of qPCR primer pairs is listed in Table S2.

### 4.15 Statistical Analysis

Statistical analysis was performed using *Student's t* tests, one‐way, or two‐way ANOVA. The data were indicated as *p < .05; **p < .01; ***p < .001. The data of statistically nonsignificant were not shown.

## CONFLICT OF INTEREST

The authors disclose no potential conflicts of interest.

## AUTHOR CONTRIBUTION

CPY designed and performed experiments in Figure 2a‐c, Figures 2e‐f, 3 and 4a‐b, Figures S3, S4 and Tables [Link], [Link], [Link], [Link]. WSY designed and performed experiments in Figures 2d, 5e‐h, and Figure S1. KHW designed and performed experiments in Figure 5a‐d. MHC designed and performed experiments in Figure 1. YCT collected mouse muscle and blood specimens and performed experiments in Figure 1a and Figure S2. KHL prepared mouse plasma specimens. FSN and JWT designed experiments related to Figure S4. WLH designed experiments related to Figure 2. MWL supervised statistical analysis. TYT generate gRNA/Cas9n plasmid. YHW contributed to the discussion of neuronal‐related research and performed experiments in Figure 4c‐g. PNW provided the human plasma specimens. MC contributed to writing of the manuscript. LKC provided the human plasma specimens and data analysis of Table 1. TFT provided mouse muscle and plasma specimens. PCC supervised CPY, WSY, and MHC. PCC and HJK contributed to all discussion and writing of the manuscript.

## ETHICAL APPROVALS

Human plasma collected from Taipei Veterans General Hospital (TVGH) was approved by the ethics committee of TVGH. The animal study was approved by Institutional Animal Care and Use Committee (IACUC) of National Yang‐Ming University, Taiwan (NYMU), and animals were kept in accordance with established regulations of the Laboratory Animal Center (LAC) at NYMU. Written informed consent was obtained from all participants in the studies.

## Data Availability

The expression level of miRNAs which analyzed with small RNA sequencing is calculated as read per millions of mapped reads (RPM) and listed in http://dx.doi.org/10.17632/972ty8mx2m.1
