# Supplementary figures and images for: Muscle atrophy‐related myotube‐derived exosomal microRNA in neuronal dysfunction: Targeting both coding and long noncoding RNAs
Source: Aging Cell. 2020 Mar 31;19(5):e13107. doi: 10.1111/acel.13107 (PMC7253071; doi:10.1111/acel.13107)

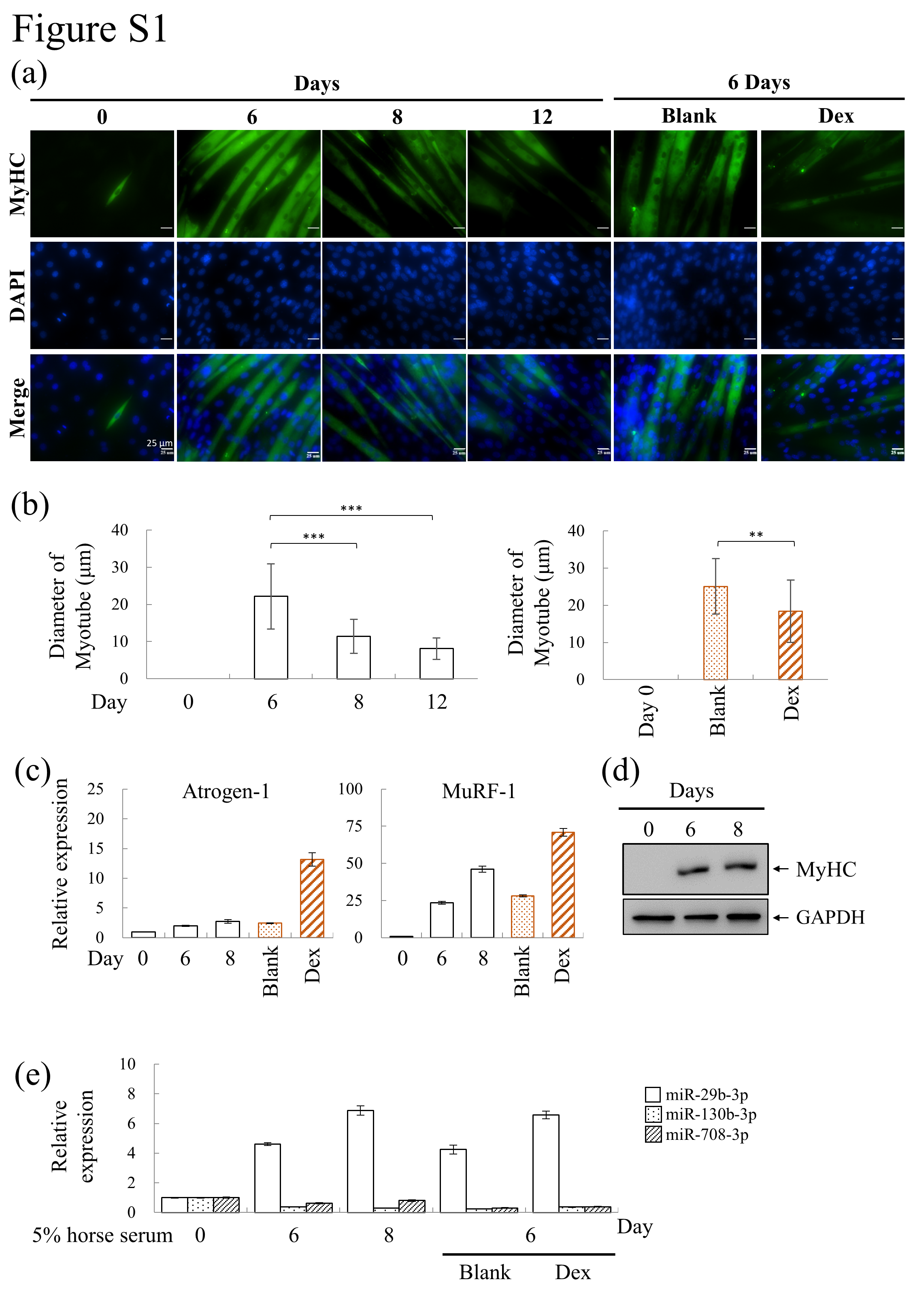

Supplement: Supplementary file 1 [file ACEL-19-e13107-s001.TIF]

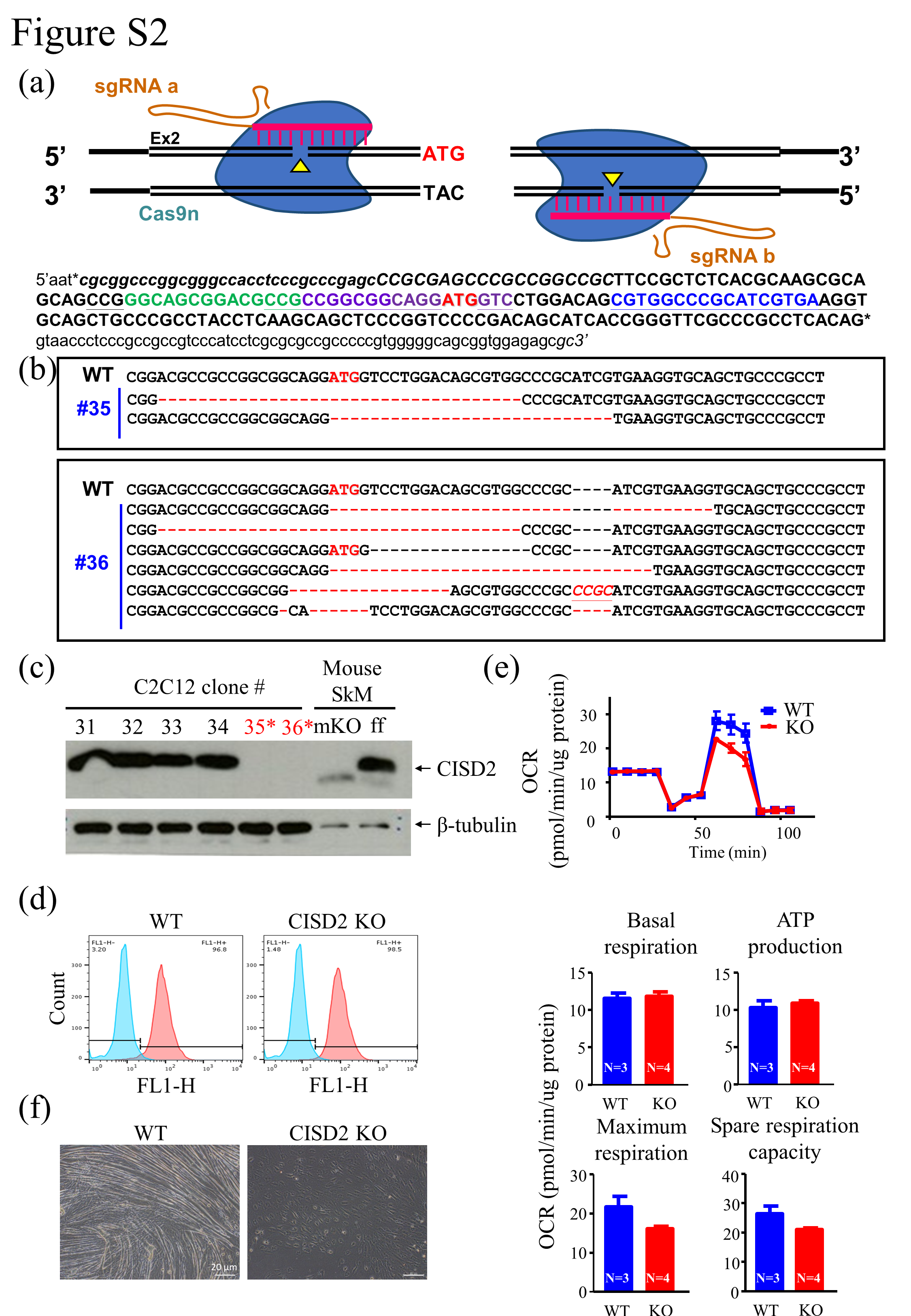

Supplement: Supplementary file 2 [file ACEL-19-e13107-s002.TIF]

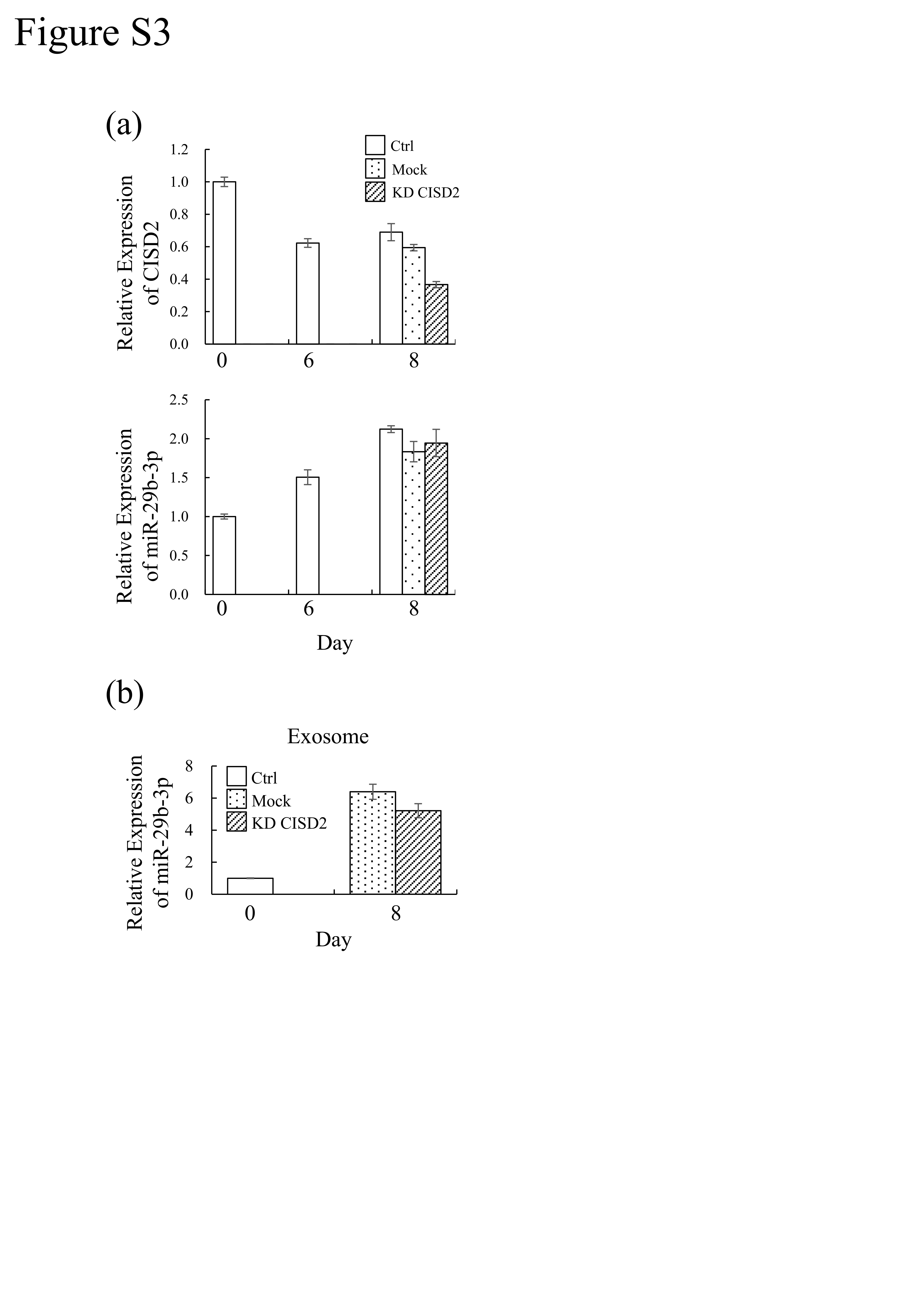

Supplement: Supplementary file 3 [file ACEL-19-e13107-s003.tif]

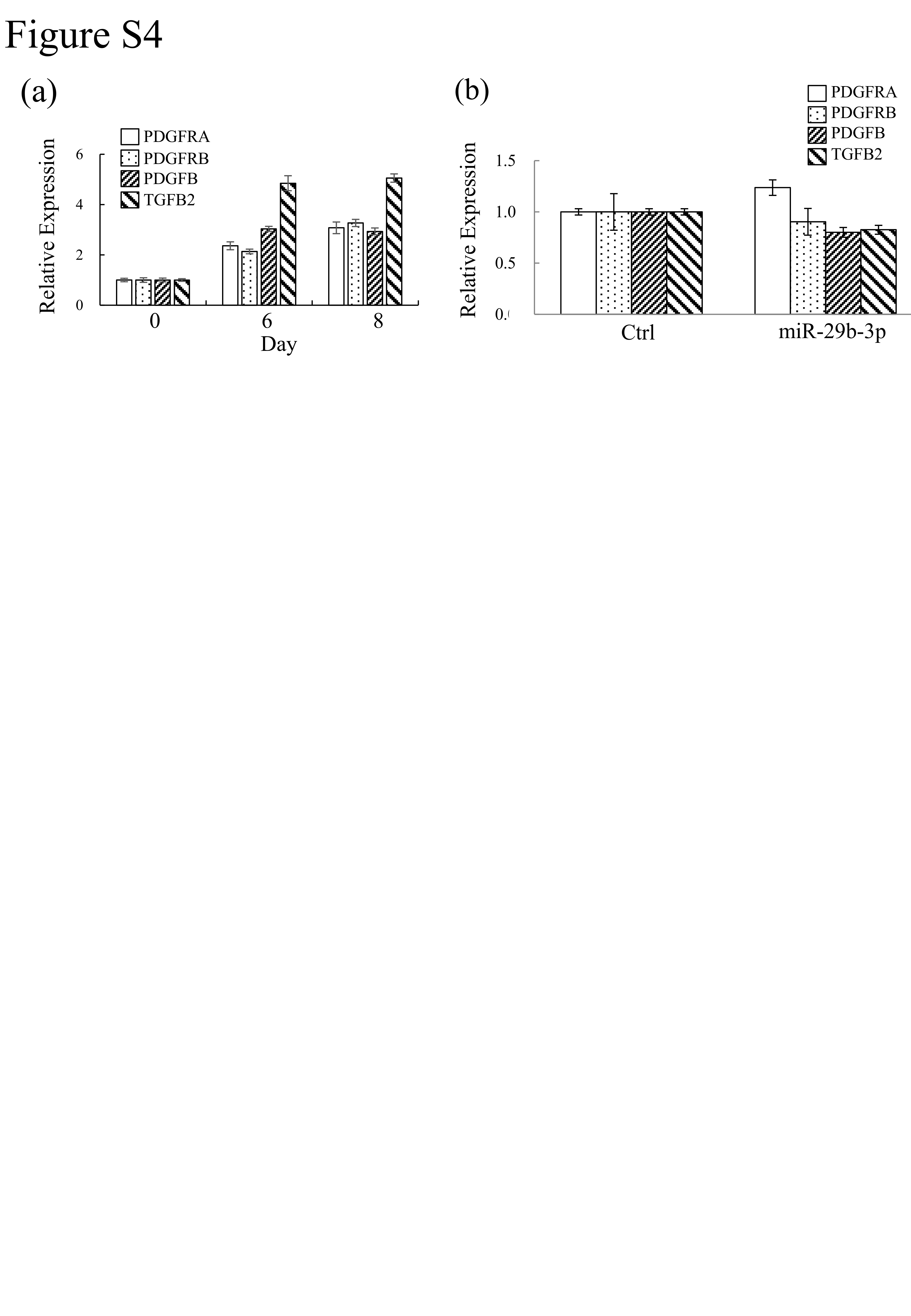

Supplement: Supplementary file 4 [file ACEL-19-e13107-s004.tif]

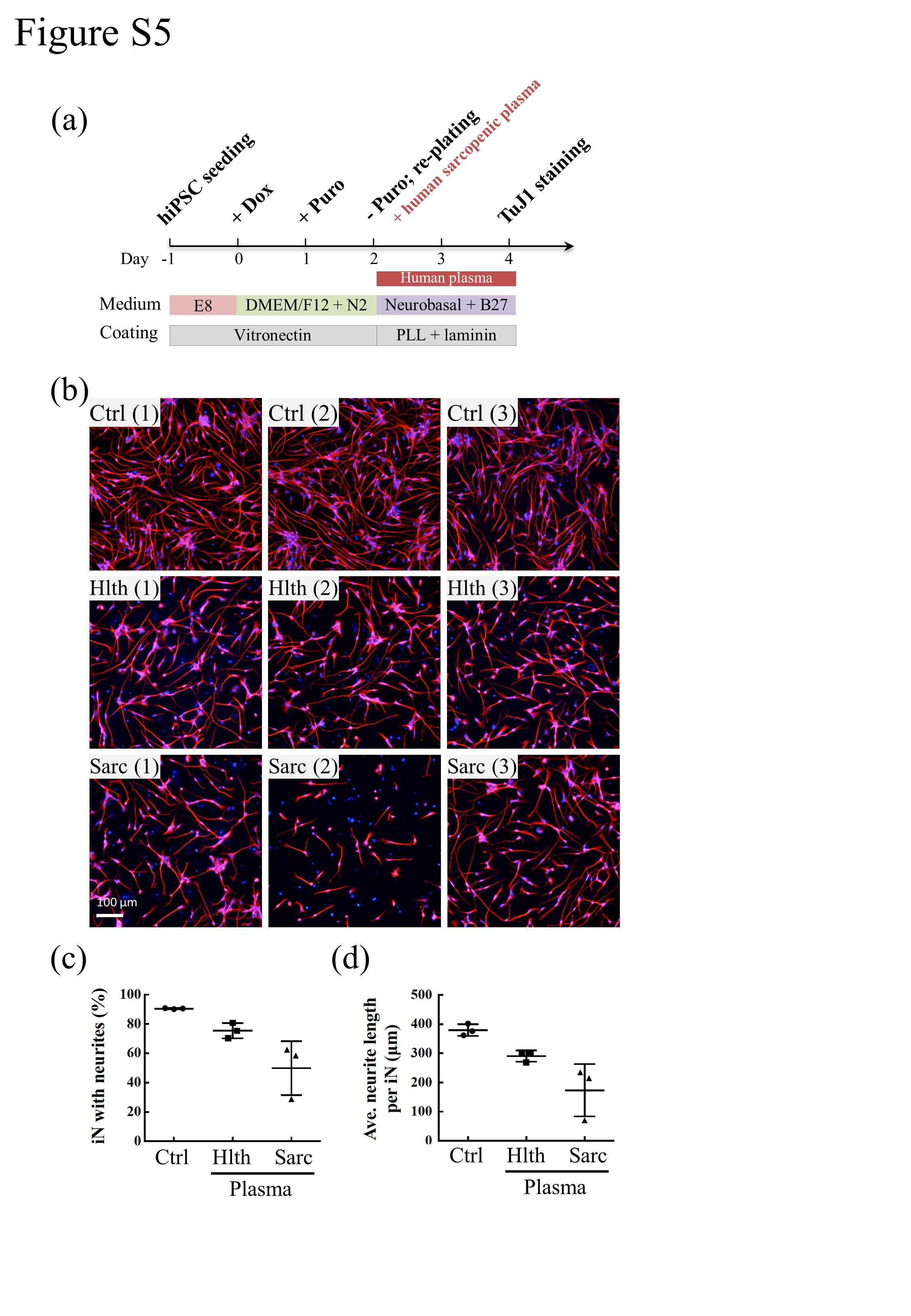

Supplement: Supplementary file 5 [file ACEL-19-e13107-s005.TIF]

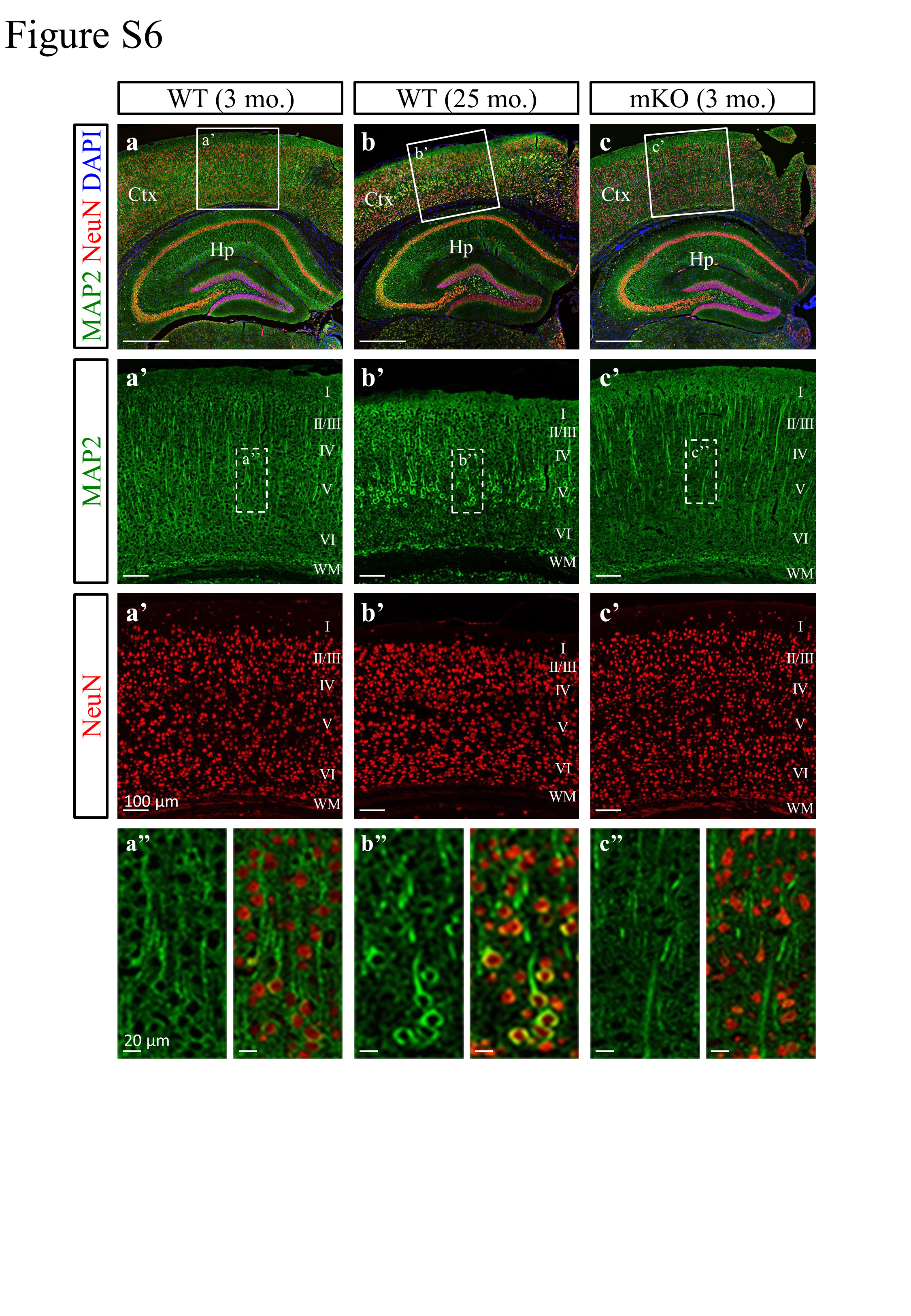

Supplement: Supplementary file 6 [file ACEL-19-e13107-s006.tif]

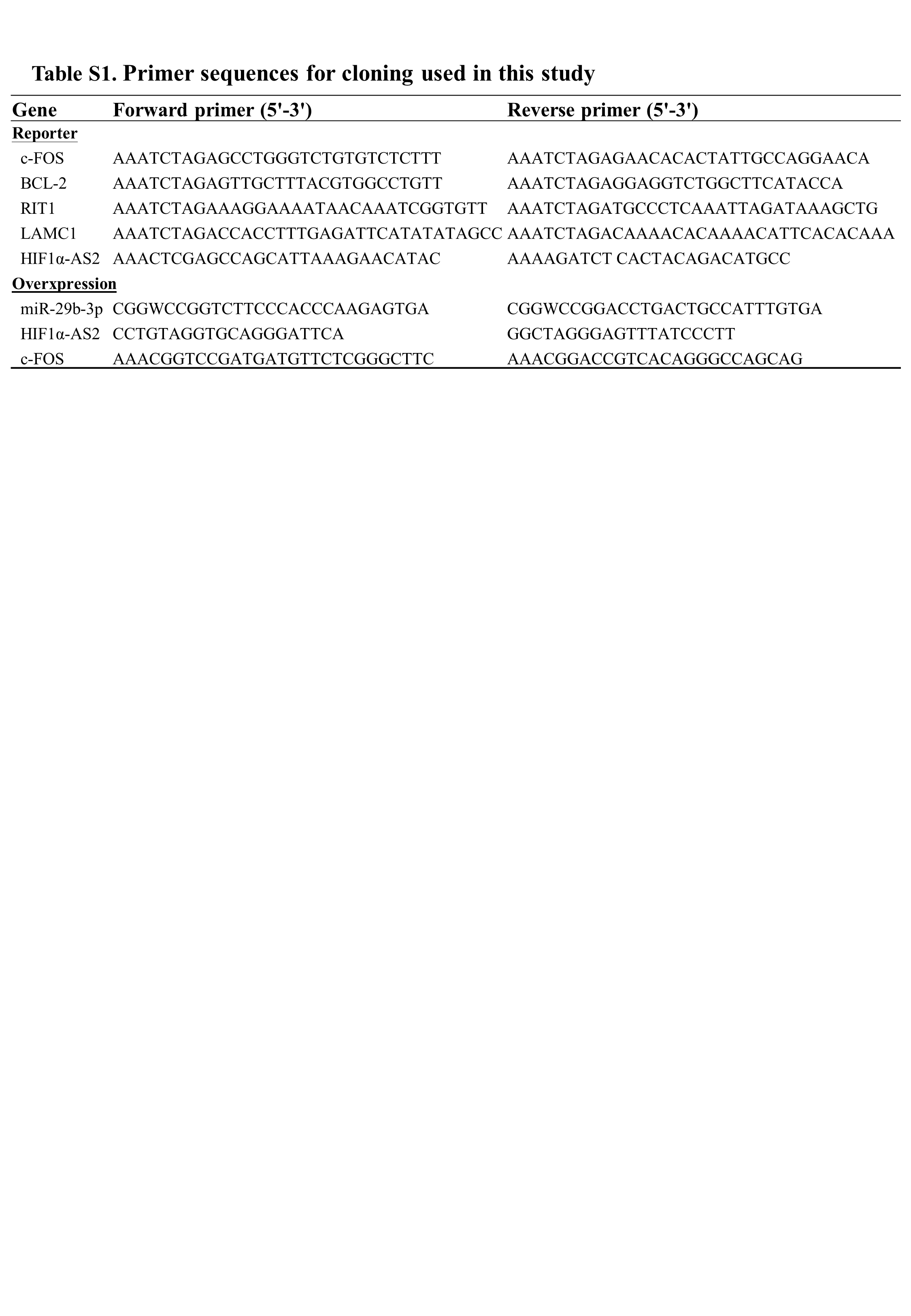

Supplement: Supplementary file 7 [file ACEL-19-e13107-s007.pdf]

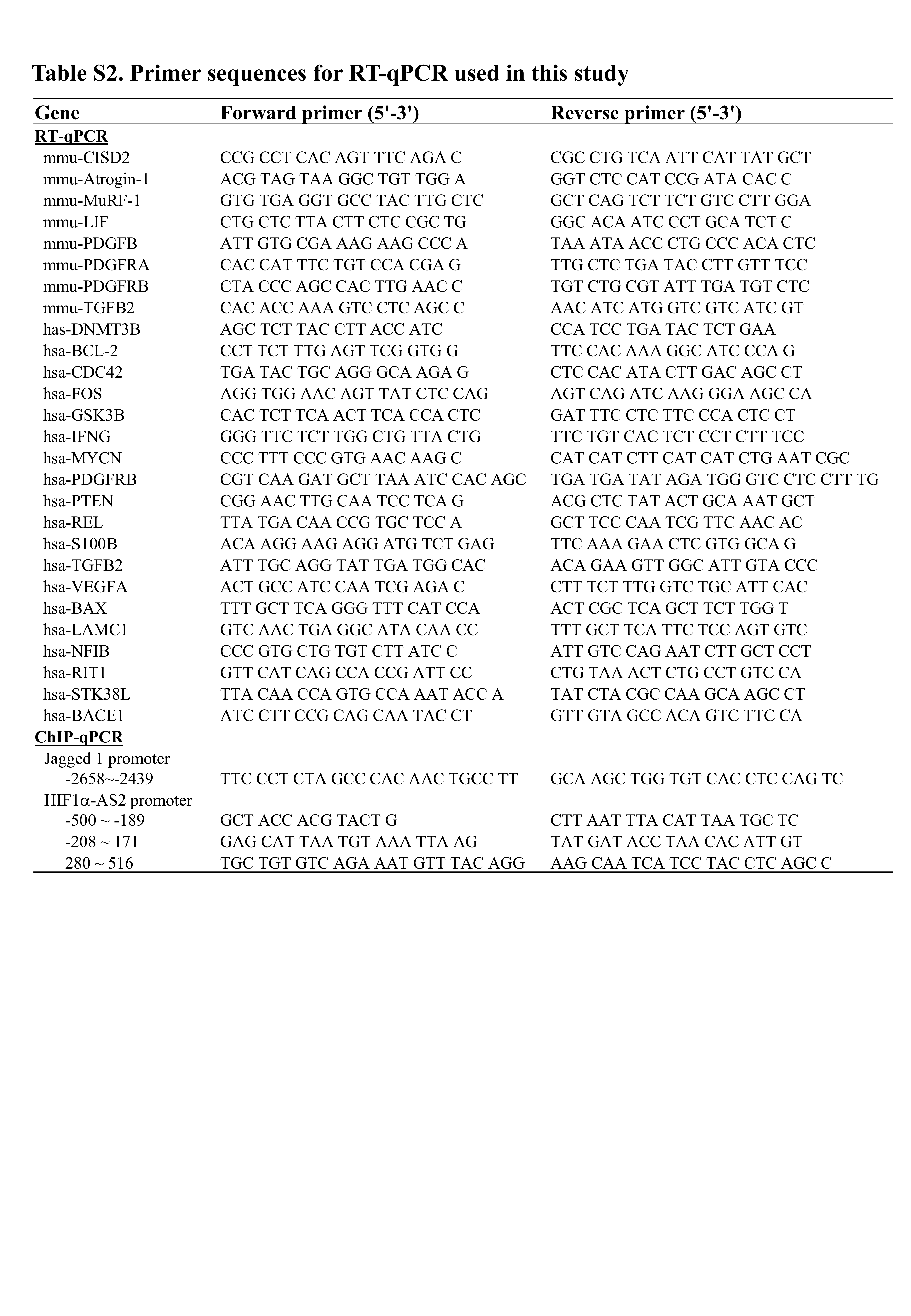

Supplement: Supplementary file 8 [file ACEL-19-e13107-s008.pdf]

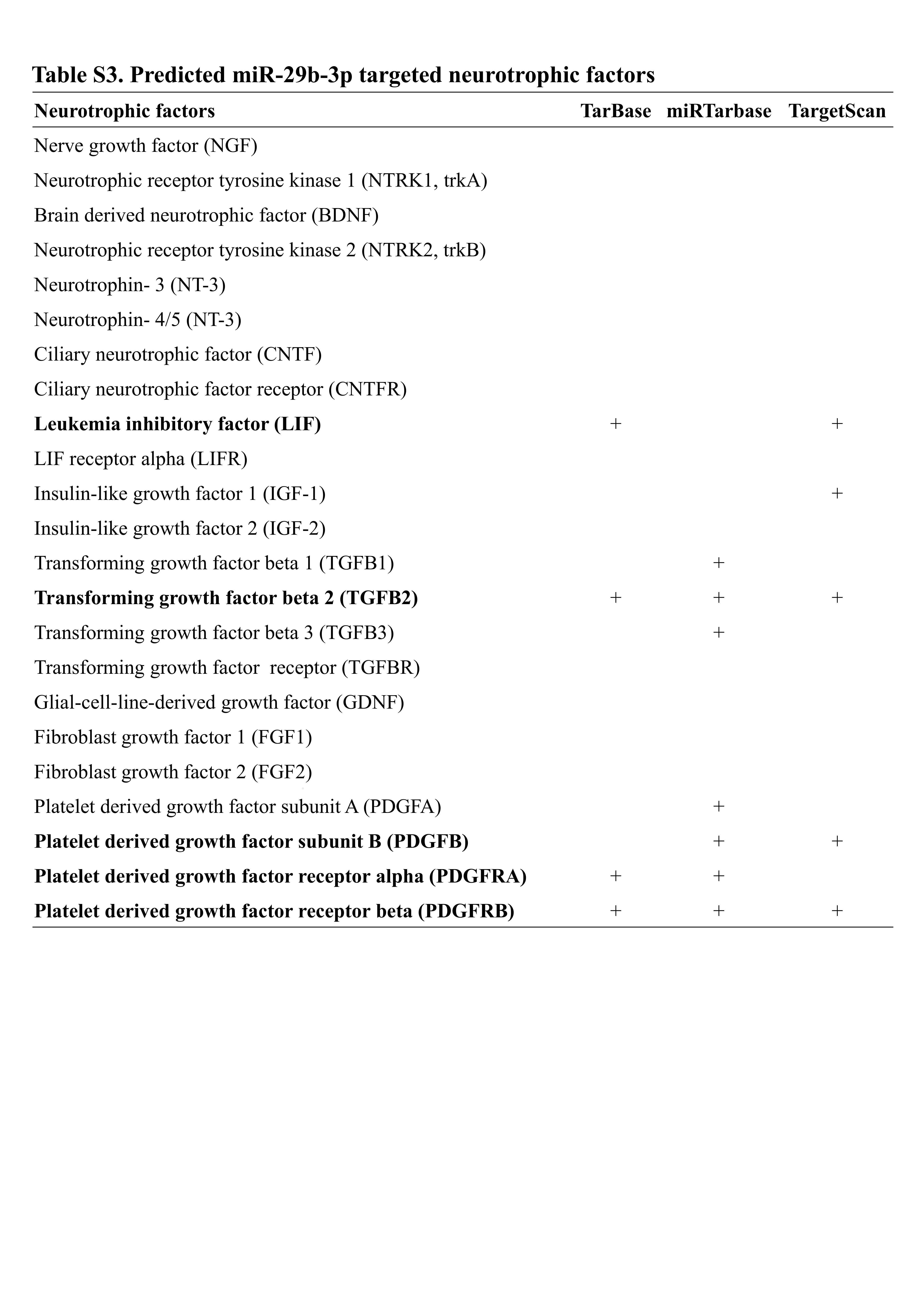

Supplement: Supplementary file 9 [file ACEL-19-e13107-s009.pdf]

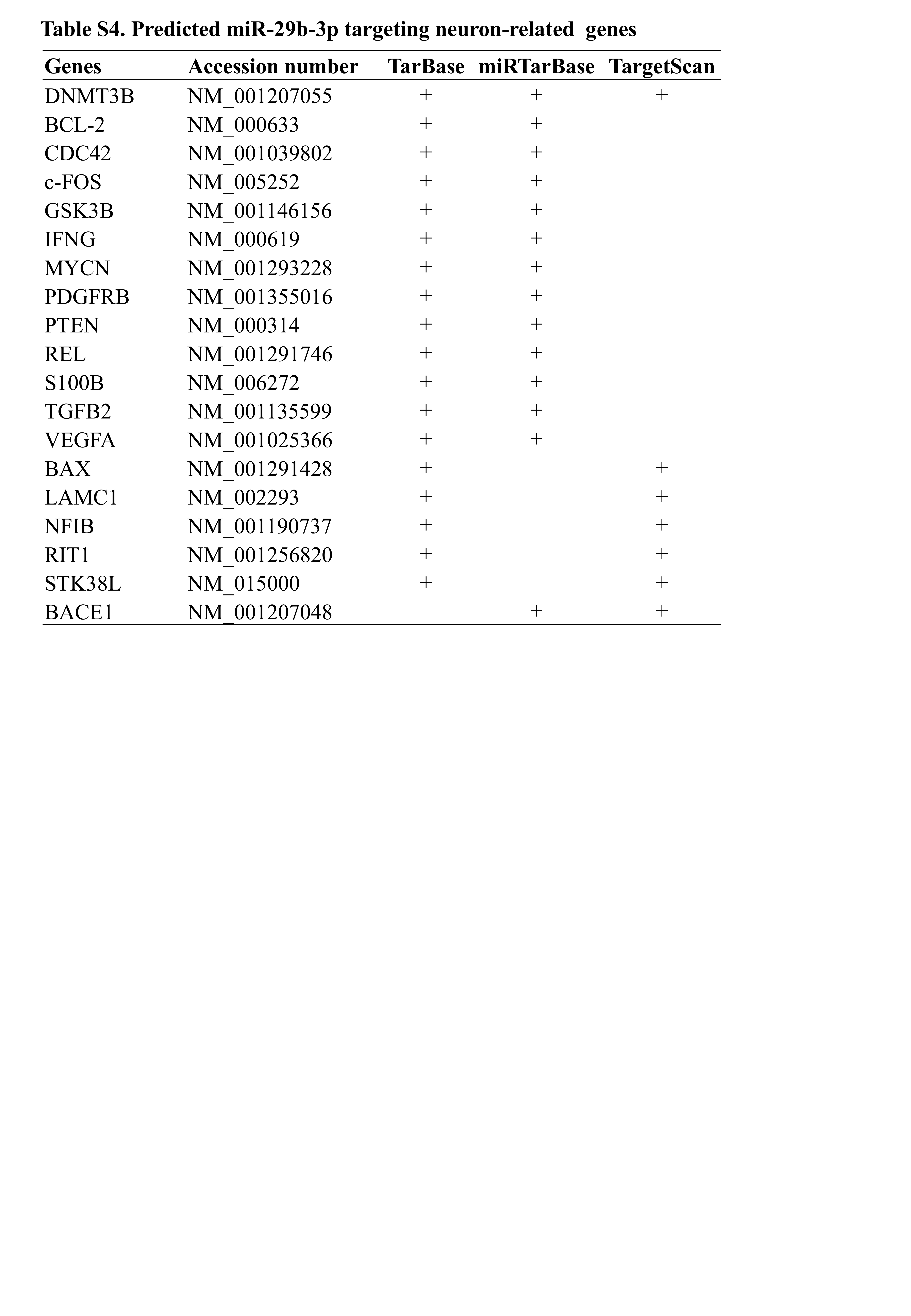

Supplement: Supplementary file 10 [file ACEL-19-e13107-s010.pdf]
