# Supplementary material for: Muscle atrophy‐related myotube‐derived exosomal microRNA in neuronal dysfunction: Targeting both coding and long noncoding RNAs
Source: Aging Cell. 2020 Mar 31;19(5):e13107. doi: 10.1111/acel.13107 (PMC7253071; doi:10.1111/acel.13107)
Supplement: Supplementary file 11 [file ACEL-19-e13107-s011.docx]

**Supporting Methods**

**Cell Culture and Plasmid**

The C2C12, and 293T cell lines were maintained in Dulbecco's Modified Eagle Medium (DMEM) (Gibco^®^, USA) supplemented with 10% fetal bovine serum (FBS) (Gibco^®^). The SH-SY5Y cell line was maintained in DMEM/F-12 (Gibco^®^) supplemented with 15% FBS. Human iPSCs were maintained on vitronectin (Gibco^®^) coated dishes in Essential 8™ (E8) medium (Gibco^®^). Cells were maintained at 37°C under a humidified 5% CO_2_ atmosphere. For SH-SY5Y differentiation, cells were treated with 10 µM retinoic acid (RA) for 72 hours. For C2C12 differentiation, cells with 80% confluence were treated with 5% exosome-free horse serum containing DMEM for 6 days and up to 12 days. The medium was changed every other day.

SH-SY5Y cells stably expressing HIF1α-AS2, SH-SY5Y-HIF1α-AS2, was generated by transfecting cells with a HIF1α-AS2 expression plasmid, pCI-HIF1α-AS2, by electroporation at 1.1 kV for 50 milliseconds, followed by 0.5 μg/ml neomycin (Cyrusbioscience) selection for 2 weeks. CISD2 KO C2C12 cell line was generated using CRISPR/Cas9n (D10A) system. The guide RNA (gRNA) of mouse CISD2 was designed by Optimized CRISPR Design (http://crispr.mit.edu). C2C12 cells were transfected with gRNA/Cas9n plasmid that also expresses EGFP and DsRed. 48 hours after transfection, GFP and DsRed double-positive cells were sorted by using FACSAria IIu (BD Bioscience). Candidate clones were tested for CISD2 knockout by immunoblotting. Deletion of CISD2 exon 1 was confirmed by polymerase chain reaction (PCR) (5’-GACGTCAAGGGTCGAAAGCGGC-3’; 5’-GATGCATGCGACAGTGAGT TCAG-3’) followed by sequencing. HiPSC-derived neurons (iNs) were generated using a published protocol (Zhang et al., 2013) with modifications. Briefly, hiPSCs that had been infected with FUW-rtTA and pTetO-NG2-puro were induced to neuronal differentiation by doxycycline (Dox, 2 mg/L) treatment. After one day, cells were subjected to a puromycin selection (1 mg/L) for 24 hours. Following selection, transduced cells were replated (2.6x104 cells/cm2) in neurobasal medium supplemented with B27/GlutaMAX (Invitrogen) containing BDNF and NT-3.

Luciferase reporter plasmids were constructed by cloning the predicted miR-29b-3p binding site on the 3’-UTR of c-FOS, BCL-2, RIT1 and LAMC1 into pGL3-Basic plasmid (Promega) using the specific primer pairs. Human HIF1α-AS2 promoter was also cloned into pGL3-Basic plasmid using specific primers pair. For overexpression experiments, a genomic DNA fragment containing the precursor sequence of miR-29b-3p was cloned into pLenti4-CMV/TO plasmid using miR-29b-3p primer pair. Human HIF1α-AS2 cDNA (2051 bps) was cloned into pCI-Neo plasmid (Promega) using HIF1α-AS2 specific primers pairs. c-FOS cDNA (1143 bps) was cloned into pcDNA3-Flag plasmid using c-FOS specific primers pair. The sequences of the primer pairs used are listed in Table S1.

**RNA extraction and reverse transcription and quantitative polymerase chain reaction (RT-qPCR)**

Mouse muscle tissues were homogenized in 2 ml of QIAzol Lysis Reagent by Precellys^®^ Tissue Homogenizing Mixed Beads Kit (Bertin) using four 15 second cycles of homogenization at 6,500 rpm. The homogenates were centrifuged at 12,000 *g* for 10 min at 4^o^C. The supernatants were collected, and total RNA was isolated using miRNeasy Mini Kit (Qiagen) according to the manufacturer's instructions. Total RNAs from cell lines were also isolated by using miRNeasy Mini Kit. Mouse plasma and human plasma were subjected to plasma RNA purification using miRNeasy Serum/Plasma Kit (Qiagen) following the manufacturer's instructions.

For miRNA detection, 0.1 µg of total RNA was reverse transcribed using miRCURY LNA miRNA PCR Starter kit (EXIQON) and detected by SYBR® Green Master Mix (BioRad) using CFX connect^TM^ real-time PCR detection system (Bio-Rad, Hercules, CA, USA). The expression of miRNAs was normalized to UniSp6 (sp6), spike-in control small nuclear RNA.

For mRNA detection, 0.5 µg of total RNA was used for RT with Oligo(dT)_12-18_ using SuperScript^III^ RT (Invitrogen). Sequence of primer pairs are listed in Table S2. For LncRNA qPCR Array, 2 µg of total RNA was used for RT using Maxima First Strand cDNA Synthesis Kit (Thermo). Gene expression was normalized to GAPDH.

**Small RNA sequencing (smRNA-seq) and data analysis**

Total RNAs purified from mouse femoris were sequenced using Illumina Genome Analyzer II*_X_* (Illumina, San Diego, CA, USA) following the standard manufacturer’s procedure. Expression levels of miRNAs were calculated and presented as read per millions of mapped reads (RPM). TarBase v7.0 (http://diana.imis.athena-innovation.gr/DianaTools/index.php?r=tarbase/index), miRTar (http://mirtar.mbc.nctu.edu.tw/human/) and TargetScan 7.0 (http://www.targetscan.org/vert_72/) were used to predict target genes and binding sites of miR-29b-3p.

**Isolation of exosomes**

For miRNA detection, 1.5 ml supernatants from C2C12 myotubes were centrifuged at 2,000 *g* for 30 min at 4^o^C to pellet dead cells. 1 ml of supernatant was transferred into a new Eppendorf, 50% volume of Total Exosome Isolation Reagent (Invitrogen) was added, mixed by vortexing for 30 seconds, and followed by incubation overnight at 4^o^C. The following day, exosome samples were centrifuged at 10,000 *g* for 1 hour at 4^o^C. The exosome pellet was solubilized in 1 ml QIAzol Lysis Reagent (Qiagen) and the total exosomal RNA was purified using miRNeasy Mini Kit following the manufacturer's instructions.

To prepare a large quantity of exosomes, 0 and 8 days after horse serum-induced differentiation of C2C12, supernatants were harvested and centrifuged at 2,000 *g* for 20 min at 4^o^C to pellet cellular contaminants, followed by another centrifugation at 10,000 *g* for 30 min at 4^o^C to pellet cell debris. The supernatant was then ultracentrifuged at 100,000 *g* for 90 min at 4^o^C using the SW28 rotor (Beckman). The exosome pellets were washed twice with phosphate-buffered saline (PBS). The final exosome pellet was solubilized in PBS (200 ml supernatant/100 μl PBS) and stored at -80^o^C. The protein levels of exosomes were measured using Bradford Protein Assay kit (Bio-Rad) following the manufacturer’s instructions.

**Fluorescent labeling and transfer of exosomes**

Exosomes were labeled with lipophilic cell tracking dye PKH26 (Sigma) at a final concentration of 2 μM and incubated at 37^o^C for 5 min. The reaction was stopped by adding an equal volume of 1% BSA/PBS and incubated for an additional 5 min. 20 μg/ml of PKH26-labeled exosomes were co-cultured with SH-SY5Y cells. 24 hours after incubation, cells were washed with PBS, fixed by 4% paraformaldehyde for 15 min at room temperature (RT), stained with Hoechst33342 for 5 min at RT and viewed at 63x using the confocal microscope (Zeiss, LSM880). Images were merged using ZEM (Zeiss) software.

Thunder Imager 3D Live Cell (Leica Microsystems).

**Luciferase reporter assays**

Reporter assays were performed by transiently co-transfecting 293T cells with pGL3-Luc and pRL-TK-Renilla reporter plasmids (Promega), and pLenti4-CMV/TO-miR-29b-3p using TransFectin^TM^ Lipid Reagent (Bio-Rad). 48 hours after transfection, total cell lysates (TCLs) were collected and luciferase activities were measured by Dual-Luciferase Reporter Assay System (Promega), according to the manufacturer's instructions. Renilla luciferase activity was used to normalize transfection efficiency.

**Mitochondrial staining and oxygen consumption assay**

For mitochondrial staining, control and CISD2 KO C2C12 cells were collected by centrifugation, washed and then resuspended in PBS containing 20 nM cardiolipin-sensitive probe 10-nonyl-acridine orange (NAO) (Molecular Probes) for 10 min at RT. Fluorescence was subsequently measured by FACSCalibur (BD Biosciences) using the Fl-1 channel.

For oxygen consumption assays, control and CISD2 KO C2C12 cells were seeded in 24-well plate (Seahorse Bioscience) approximately 20 hours before the analysis. One hour before the experiment, the culture medium was replaced by unbuffered DMEM (pH 7.4). The oxygen consumption rate (OCR) was measured using the Seahorse XF24 (Seahorse Bioscience) according to the manufacturer’s instructions. The oxygen concentration was measured before and after adding oligomycin (final concentration 1 μM), the mitochondrial uncoupler FCCP (1 μM) and antimycin A (1 μM). Following the assay, the cells were lysed and the total protein concentration was measured. The amount of total protein was used for the normalization of OCR.

**Immunofluorescence (IF) staining**

C2C12, SH-SY5Y and hiPSC lines (1x10^5^ cells/well) were seeded on the coverslips in 6 well plates. C2C12 and hiPS cells were fixed by 4% paraformaldehyde (PFA) for 15 min at RT, permeabilized with 0.5% Triton X-100 in PBS for 15 min and blocked with 1% BSA/PBS for 1 hour at RT. C2C12 cells were then incubated with primary anti-MyHC antibody (1:100, DSHB) in 5% BSA at 4^o^C overnight, washed three times with PBS and incubated with Alexa Fluor^®^ 488 AffiniPure Goat Anti-Mouse IgG (H+L) (Jackson) for 1 hour at RT, followed by staining with Hoechst33342 for 5 min at RT. hiPSCs were incubated with anti-MAP2 (1:1000, Millipore) and anti-neurofilament (Smi-312, 1:1000, BioLegend) antibodies in 5% BSA at 4^o^C overnight, washed three times with PBS and incubated with Fluor^®^ 488 AffiniPure Goat Anti-Rabbit IgG (H+L) (Invitrogen) for 1 hour at RT, followed by staining with DAPI for 5 min at RT. SH-SY5Y cells were fixed by 4% PFA and stained by Neurite Outgrowth Staining kit (Thermo) according to the manufacturer's instructions. Images were visualized by Leica DMI1000 B in 5 to 20 randomly chosen fields (40x). Diameter of myotubes and neurite length was measured using Image J and MetaMorph, respectively.

Wild-type or CISD2 mKO mice were anesthetized and sacrificed at different time points. Their brains were rapidly removed, immersed in 10% formalin and then paraffin embedded. Coronal sections (5 μm thick) were cut across the dorsal hippocampus, mounted onto polarized slides and stored at -80^o^C. For IF staining, sections were dewaxed and rehydratated and antigens were unmasked using a commercially available kit (Unmasker, Diapath). Tissues were permeabilized with 0.5% Triton X-100 in PBS for 10 min at RT, washed twice in PBS, blocked with 5% BSA and 5% goat serum for 30 min at RT and then incubated in humid atmosphere with the primary anti-NeuN and anti-MAP2 (Millipore Bioscience Research Reagents) antibodies at 4°C overnight. After washing, slices were incubated with Alexa Fluor^®^ 488 or 568 AffiniPure Goat Anti-Mouse IgG (H+L) (Jackson) for 1 hour at RT, followed by staining with DAPI. Images were visualized by Leica THUNDER Imager 3D Live Cell.

**Immunoblotting assay**

TCLs were prepared using RIPA lysis buffer (50 mM Tris, pH 7.4, 150 mM NaCl, 1% NP-40, 1% sodium deoxycholate, 1 mM EDTA, ddH_2_O), separated by SDS-PAGE and transferred to PVDF membranes using semidry transfer system (Bio-Rad). The membranes were blocked with 5% milk or 5% BSA for 1 hour at RT, followed by incubation overnight with primary antibody as follows: anti-MyHC (DSHB), anti-CISD2, anti-CD81 (GeneTex), anti-CD9 (Abcam) and anti-calreticulin (Abcam). Anti-GAPDH (GeneTex), anti-β-actin (GeneTex), and anti-β-tubulin (GeneTex) were used as loading controls.
