# Supplementary material for: Muscle atrophy‐related myotube‐derived exosomal microRNA in neuronal dysfunction: Targeting both coding and long noncoding RNAs
Source: Aging Cell. 2020 Mar 31;19(5):e13107. doi: 10.1111/acel.13107 (PMC7253071; doi:10.1111/acel.13107)
Supplement: Supplementary file 12 [file ACEL-19-e13107-s012.doc]

**FIGURE S1.** miR-29b-3p is increased in long-term differentiation-induced C2C12 myotubes. (a) To induce myotube differentiation, C2C12 myoblasts at 80% confluency were treated with 5% horse serum for 12 days. Representative images (40x) of immunofluorescent staining for C2C12 myotubes showing that 8 and 12 days of differentiation results in a muscle atrophy phenotype as evidenced by the reduction of myotube diameter. 10 μM Dex treatment for 24 hours was used to provide an unbiased evaluation of C2C12 myotube atrophy. MyHC, anti-myosin heavy chain; DAPI, nuclear counterstain. (b) Quantification of myotube diameter in (a). (c) RT-qPCR analysis of Atrogin-1 and MuRF-1 expression in C2C12 myotubes in (a). (d) Immunoblotting of MyHC in short (6 days)- and long (8 days)-term differentiated C2C12 myotubes. (e) RT-qPCR analysis showing a significant increase of miR-29b-3p, but not miR-708-3p and miR-130b-3p, in long-term differentiated and Dex-treated C2C12 myotubes. Blank, solvent control (a, b, c, e). Error bars show SD. ***p*<0.01, ****p*<0.001 by One-way ANOVA (b, left panel) and *Student’s-t* test (b, right panel).

**FIGURE S2.** CRISPR/Cas9n system for CISD2 gene editing in C2C12 myoblasts. (a) Schematic illustrating DNA double-stranded breaks using a pair of Cas9 D10A nickases (Cas9n). The sequence of sgRNAa1 (green), sgRNAa2 (purple), and sgRNAb (blue) is shown. (b) The knockout clones (#35 and #36) were confirmed by sequencing. Deleted sequences are indicted by dotted lines. (c) Immunoblotting of CISD2 in different CISD2 KO clones. (d) Histogram of NAO fluorescence intensity of wild-type (WT) and CISD2 KO C2C12 cells is illustrated. (e) The oxygen consumption rate (OCR) was measured in C2C12 myoblasts of CISD2 WT and KO. (f) Representative images (10x magnification) of 5% horse serum induced differentiated CISD2 WT and KO C2C12 cells.

**FIGURE S3.** miR-29b-3p is not controlled by CISD2. (a) Short-term differentiated C2C12 myotubes were transiently transduced with lentivirus carrying control or pLKO.1-shCISD2 (TRCN0000183937) vector. 48 hours after transduction, knockdown of CISD2 (upper panel) and expression of miR-29b-3p (lower panel) was detected by RT-qPCR. (b) Supernatants from control and CISD2 knockdown cells were collected and subjected to exosome RNA purification using Total Exosome Isolation Reagent. RT-qPCR analysis showed a similar expression profile of miR-29b-3p. Error bars show SD.

**FIGURE S4.** miR-29b-3p does not target neurotrophic factors. (a) RT-qPCR analysis demonstrating similar expression profiles of neurotrophic factors in short- and long-term differentiated C2C12 myotubes. (b) C2C12 cells were transiently transduced with lentivirus carrying control or pLenti4-CMV/TO-miR-29b-3p vector. 48 hours after transduction, the levels of neurotrophic factors were determined by RT-qPCR. Error bars show SD.

**FIGURE S5.** Human sarcopenia plasma represses iNs cell differentiation. (a) Schematic depiction of the experimental procedure. hiPSCs were first induced for neuronal differentiation by NG2 expression. Two days after induction, the iNs were cultured in neurobasal medium (control, Ctrl) supplemented with 20% human plasma from 3 elderly subjects wirh sarcopenia (Sarc1-3) or 3 healthy controls (Hlth1-3) for 48 hours, and then immunostained with anti-TuJ1 (red) antibody and DAPI (blue). (b) Representative images of iNs stained as described in (a). Scale bar, 100 μm. (c) Quantification of the percentage of iN cells with neurites length >2 fold longer than the cell body. The lengths of neurites marked by TuJ1 staining were quantified using Neuron J software. 6-10 fields for each condition were evaluated. (d) Quantification of the average neurite length of iN cells. 85-100 neurons from randomly selected fields for each condition were evaluated.

**FIGURE S6. Age-related dendritic changes in the mice cerebral cortex.** (A-C) Coronal sections of the brains from 3 month and 25 month old wild-type and 3 month old CISD2 mKO mice were immunostained for MAP2 (green) and NeuN (red). Cells nuclei were stained with DAPI (blue). A’-C- are higher magnifications of the white boxes in A-C; A’’-C’’ are higher magnifications of the white boxes in A’-C’. Ctx, cortex; Hp, hippocampus. Scale bar, 500 μm in A-C; 100 μm in A’-C’; 20 μm in A’’-C’’.
